# Kinesin-1 is highly flexible and adopts an open conformation in the absence of cargo

**DOI:** 10.1016/j.isci.2026.114875

**Published:** 2026-02-02

**Authors:** Evelyn R. Smith, Emma D. Turner, Mahmoud A.S. Abdelhamid, Timothy D. Craggs, Alison E. Twelvetrees

**Affiliations:** 1 Division of Neuroscience, School of Medicine and Population Health, Faculty of Health, The University of Sheffield, Glossop Road, Sheffield S10 2HQ, UK; 2 School of Mathematical and Physical Sciences, Faculty of Science, The University of Sheffield, Dainton Building, Brook Hill S3 7HF, UK; 3 Exciting Instruments, Block 5, Level 9, Pennine Five Campus, 18 Hawley Street, Sheffield City Centre, Sheffield S1 4WP, UK

**Keywords:** molecular biology, cell biology

## Abstract

Kinesin-1 is an essential anterograde microtubule motor protein. The core kinesin motor is a homodimer of two heavy chains; N-terminal motor domains hydrolyze ATP and walk along microtubules, while a long elongated coiled-coil stalk and an intrinsically disordered C-terminal tail region bind cargos. Kinesin autoinhibition is key to preventing futile ATP consumption and occurs, at least in part, through direct interactions between N-terminal motor domains and C-terminal inhibitory motifs. Despite significant advances in our understanding of kinesin walking, little is known about the kinesin-1 conformational landscape of the stalk and tail domains. Here, we apply solution-based biophysical analysis tools to study conformational changes in kinesin-1, with full rotational freedom, and in response to changes in ionic strength, mutations, and the presence of microtubules. This has allowed us to uncover the inherent flexibility in kinesin-1, which gives insights into autoinhibition and the regulation of intracellular transport.

## Highlights

• Kinesin-1 is highly flexible; it exists as a broad conformational ensemble
• Most kinesin-1 dimers in solution adopt an open conformation
• Ionic strength modulates kinesin-1 conformation in solution
• Microtubules polarize kinesin-1 conformation, suggesting facilitated autoinhibition

## Graphical abstract

**Figure:**
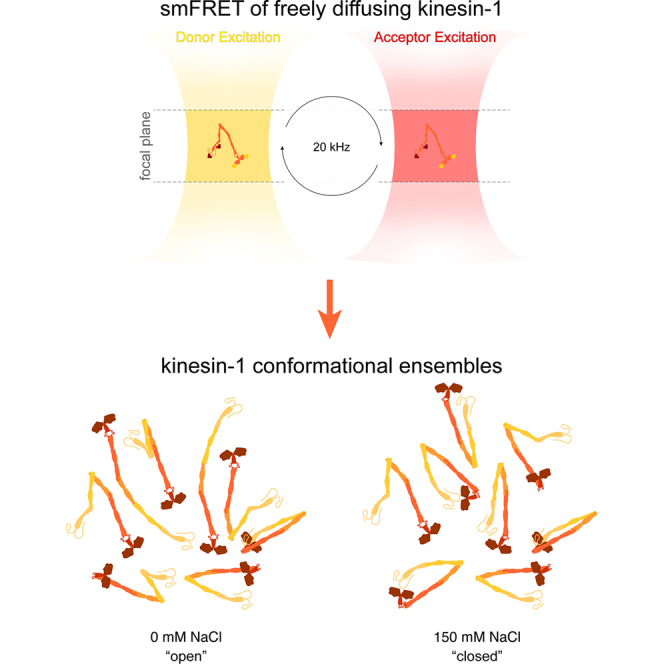

## Introduction

Kinesin-1, a ubiquitous molecular motor, plays a critical role in intracellular transport by moving many different kinds of cargo long distances along microtubule filaments. This diversity of cargos includes not only many different membrane-bound organelles^1^^,^^2^^,^^3^^,^^4^^,^^5^^,^^6^ but also RNA,^7^^,^^8^^,^^9^ cytosolic protein complexes,^10^^,^^11^ and microtubules themselves.^12^^,^^13^ While significant progress has been made toward understanding the fundamental mechanisms of how kinesin-1 turns ATP hydrolysis into motility,^14^^,^^15^^,^^16^ the regulation of its activity and its ability to interact with such a diverse range of cargo in many different local cellular environments are not fully understood. Recent studies have highlighted the importance of conformational changes in modulating kinesin-1 function.^17^^,^^18^^,^^19^ However, the complex interplay between conformational changes and microtubule interactions requires further investigation.

The core unit of kinesin-1 is a homodimer of two heavy chains; in vertebrates, there are three heavy-chain isoforms named KIF5A, B, and C. Kinesin-1 heavy chains consist of the well-conserved N-terminal motor domains, a long elongated coiled-coil stalk, and an intrinsically disordered C-terminal tail region.^14^^,^^20^ Kinesin-1 homodimers can be supplemented by a pair of kinesin light chains (KLCs 1–4 in vertebrates), but light-chain-independent functions of kinesin-1 are known,^2^^,^^8^^,^^12^ and several studies indicate that kinesin light chains are not required for motility *in vitro*.^21^^,^^22^^,^^23^ Indeed, both light-chain-bound and unbound populations can be purified from the brain,^24^ and protein copy number estimates indicate a consistent molar excess of heavy chains compared to light chains.^25^ Consequently, the kinesin-1 heavy chains alone are sufficient to recapitulate both autoinhibition and motility.

Basic “open” and “closed” conformational states of the kinesin-1 have been defined almost since its discovery.^26^^,^^27^ Kinesin-1 is a microtubule-stimulated ATPase,^28^ where autoinhibition prevents futile ATP consumption when kinesin is not bound to cargo. The tail of kinesin contains a conserved IAK motif that is important for autoinhibition via a “head-to-tail” interaction where the IAK motif docks between the motor domains^29^^,^^30^^,^^31^^,^^32^; thus, the closed conformation of kinesin has become synonymous with an autoinhibited molecule. However, recent evidence suggests the IAK motif is not the sole component of autoinhibition, and other interactions may also be in play.^18^^,^^33^^,^^34^ By definition, autoinhibited conformations must be highly unfavorable when kinesin-1 is actively walking along microtubules, and so walking kinesin is synonymous with an open conformation. Beyond this, the conformational landscape of kinesin-1 is poorly defined, including the conformational changes required to allow walking along microtubules and whether the open conformation is a single conformer or represents a broader range of related structures. A binary open/closed model implies a rapid interconversion between these two states, although it is currently unknown if this is the case. Part of the reason that the conformational landscape of kinesin-1 remains poorly defined is likely the paucity of high-resolution structural data for kinesin-1 beyond the well characterized motor domains. This in turn is compounded by commonly used methods in the field, such as single-molecule walking assays, giving information only on the end product of these complex events as kinesin walks along microtubules. Consequently, the conformational changes within the stalk and tail of kinesin are often assumed rather than measured directly.

In order to better understand the ability of kinesin-1 to carry out such a diverse range of transport functions, we sought to establish an experimental pipeline that could capture the kinesin-1 conformational landscape. We wanted to be able to study freely diffusing molecules, as immobilization of kinesin can cause aberrant activation through nonspecific interactions with the kinesin tail (a property leveraged for some motility studies of full-length kinesin^33^^,^^35^). We also wanted the ability to study kinesin in response to changes in both external and internal conditions; external conditions include not only factors such as ionic strength and pH but also the presence or absence of microtubules and cargos, while the internal state of the protein includes mutations or posttranslational modifications. To this end, we applied both single-molecule fluorescence resonance energy transfer (smFRET) and flow-induced dispersion analysis (FIDA) to the study of kinesin-1. smFRET in particular is emerging as a powerful tool for monitoring conformational changes, as the single-molecule resolution provides information on multiple conformers, rather than averaged ensemble measurements of populations.^36^^,^^37^

By utilizing self-labeling enzymes and cell-permeable fluorophores, we were able to probe the conformational landscape of kinesin-1 and its response to different external (ionic strength and microtubules) and internal (mutants) conditions. Given that dimers of kinesin-1 heavy chains recapitulate the two key features of kinesin, motility and autoinhibition, we reasoned that key conformational changes could be captured with heavy chains alone. Our findings reveal the dynamic conformational landscape of kinesin-1 dimers, with ionic strength and microtubule binding playing crucial roles in modulating the equilibrium between open and closed states. These insights provide a deeper understanding of the molecular mechanisms underlying kinesin-1 function and have implications for understanding the regulation of intracellular transport.

## Results

### Self-labeling enzymes and cell-permeable fluorophores are suitable for smFRET

To measure conformational changes in kinesin-1, we chose to use the self-labeling enzymes CLIP and SNAP to position fluorophores at the N and C termini of kinesin-1. Although dependent on the fluorophores in use, the typical dynamic range for fluorescence resonance energy transfer (FRET) occurs when fluorophores are 3–10 nm apart. The extended conformation of kinesin is thought to be between 60 and 80 nm,^26^^,^^38^^,^^39^ greatly exceeding this limit, while the autoinhibited conformation should bring the N and C termini into close proximity,^29^^,^^30^ creating a signal change upon closing of kinesin.^40^ As of yet, there are no experimentally determined high-resolution structures of the kinesin-1 stalk region that would allow the considered positioning of fluorophores within the coiled coils by direct labeling approaches. Consequently, self-labeling enzymes allow the conjugation of bright fluorophores to proteins, without the additional challenges imposed by the maturation of fluorescent proteins and their concomitant spectra.^41^ We chose to pair SNAP and CLIP, as they are matched in size (19.4 kDa) and are smaller than other self-labeling enzymes such as the HaloTag (33 kDa); as FRET is highly distance dependent, this is a key advantage.

**Figure 1:**
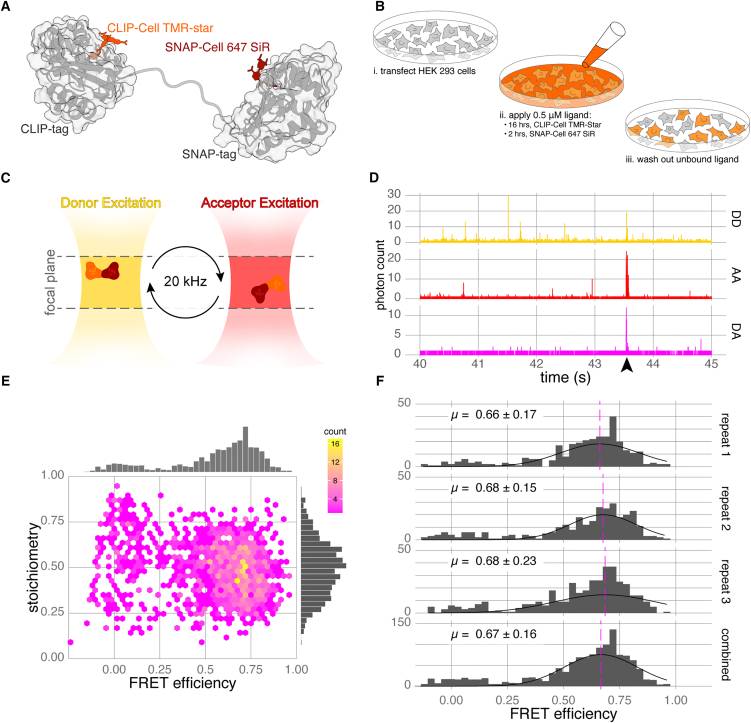
A CLIP-SNAP fusion protein is suitable for smFRET measurements using live-cell compatible fluorescent ligands (A) Schematic of the labeled CLIP-SNAP fusion protein with the flexible linker (PDB: 6Y8P). (B) Steps for sample preparation requiring optimization to minimize background contribution from unbound ligand. (C) smFRET in the confocal volume using alternating laser excitation (ALEX); only one molecule is present in the volume at any one time. Single molecules diffuse through the confocal volume (∼1 μm^3^) while being probed by ALEX cycles of donor and acceptor excitation at 20 kHz.^42^ (D) Example time traces of TMR-Star and 647-SiR-labeled CLIP-SNAP from the donor:donor (DD), acceptor:acceptor (AA), and donor:acceptor (DA) excitation:emission channels. FRET is characterized by a photon burst in all three channels (marked by arrowhead). (E) Hexbin plot of FRET efficiency versus stoichiometry for the CLIP-SNAP fusion protein expressed in HEK293 cells. Data from five biological replicates, *n* = 1389 bursts. (F) Histograms of FRET efficiency from three biological repeats demonstrating reproducibility of live-cell labeling for smFRET. Stoichiometry is gated to 0.25 and 0.75.

To test whether the CLIP-tag and SNAP-tag had the capacity to be used for smFRET experiments with readily available fluorescent ligands, both were fused with a short flexible linker to provide a high-FRET positive control (CLIP-SNAP, Figure 1A). HEK293 cells were transfected with CLIP-SNAP and labeled with the cell-permeable fluorophores CLIP-Cell TMR-Star and SNAP-Cell 647-SiR (Figure 1B); the cell lysate was collected and used directly for measurements. High-quality confocal smFRET measurements rely on the diffusion of a single molecule through the confocal volume at any one time (Figure 1C). In practice, although events are short (a few milliseconds), an observation rate of about one molecule per second avoids coincidence events of two or more molecules in the volume at a time, producing erroneous FRET measurements (Figure 1D). Any unbound ligand or other background contaminants contribute to this rate of observation, increasing acquisition time to many hours and making experiments impractical. Our preliminary results were promising and FRET bursts were observed, but the background of unbound ligands made acquisition very slow.

To eliminate background contributions from unbound fluorescent ligands, we undertook a systematic optimization of labeling for CLIP- and SNAP-tagged protein expressed in HEK293 cells, and the cell-permeable fluorophores CLIP-Cell TMR-Star and SNAP-Cell 647-SiR. This included using flow cytometry to calculate the optimal washout time for unbound ligand using untransfected cells (Figures S1A and S1B) and in-gel fluorescence of transfected cell lysate to optimize both ligand incubation time (Figures S1C and S1D) and concentration (Figures S1E and S1F). As previously shown,^43^ the labeling kinetics for CLIP-tag are slower than SNAP-tag, and our final labeling parameters were 0.5 μM of CLIP-Cell TMR-Star and SNAP-Cell 647 SiR ligands on cells for 16 and 2 h respectively, followed by 2 h in ligand-free media to wash out unbound fluorophores. We observed minimal cross-reactivity of labeling between CLIP-tag and SNAP-tag with these conditions (Figure S1G). Expressing and labeling protein in this way circumvented the need for protein purification, taking advantage of the cell membranes to act as dialysis membranes and remove unbound ligands (Figure 1B). Using in-gel fluorescence, we carried out similar experiments with the HaloTag and the TMR ligand, which showed some non-specific binding on the same timescales (Figure S1H).

Having removed contamination from the sample, the assay buffer became our biggest contributor to background—specifically BSA, regardless of source (Figure S1I). BSA is frequently added to *in vitro* assays of kinesin function as a stabilizer. We devised an in-house method to pre-bleach the BSA (see STAR Methods, Figure S1J), which eliminated this final source of contamination. However, it is still the case that any use of colored plastics in protocols (tips, tubes etc.) or other significant colored items (e.g., hair dye) is sufficient to contaminate reagents and render data acquisition impossible. Following optimization, background photon counts of diluted untransfected cell lysates derived from cells exposed to the ligand-labeling procedure were indistinguishable from the assay buffer alone (Figures S1K and S1L).

During a confocal smFRET experiment, single molecules diffuse through the confocal volume while being probed with alternating laser excitation (ALEX) for the donor and acceptor fluorophores. During ALEX, only the donor or excitation laser is on at any one time, cycling at 20 kHz; a typical molecule in the volume for 2 ms will experience 10 laser cycles. In response to excitation, each fluorescent molecule has a burst of photons (Figure 1D) that can be separated into three channels based on the excitation laser and corresponding emission wavelength. These are donor excitation and donor emission (DD); acceptor excitation and acceptor emission (AA); and donor excitation and acceptor emission (DA), also known as the FRET channel (Figure 1D). The number of photons in each of these channels is used to calculate the FRET efficiency (a measure of energy transfer from donor to acceptor fluorophore, indirectly proportional to the distance between them) and stoichiometry for that molecule (a ratio of donor to acceptor labeling). With our optimized labeling conditions, we observed robust smFRET signals of the CLIP-SNAP fusion protein after expression and dilution of lysate harvested from transfected HEK293 cells. Plotting FRET efficiency vs. stoichiometry as a frequency for all observed molecules of the CLIP-SNAP fusion protein (Figure 1E) shows a peak at 0.68 and 0.5 for FRET efficiency and stoichiometry, respectively. The stoichiometry agrees with a 1:1 labeling ratio. Furthermore, estimates of mean FRET efficiency (± standard deviation) for three independent biological replicates were 0.66 ± 0.17, 0.68 ± 0.15, and 0.68 + 0.23 (Figure 1F), demonstrating a high degree of reproducibility. Having established that CLIP-tag and SNAP-tag together with cell-permeable fluorophores are suitable for smFRET, we applied our labeling method to kinesin-1.

### HEK293 cells are a suitable expression system for full-length motor protein studies

**Figure 2:**
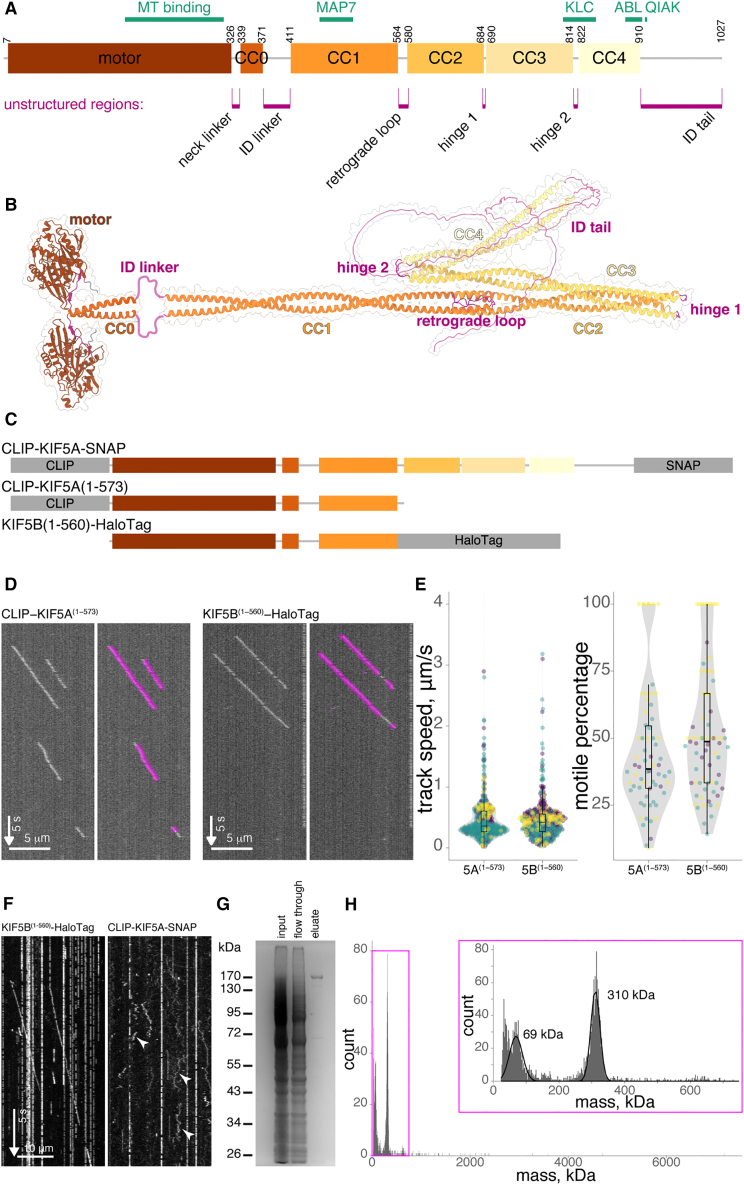
Tagged KIF5A is functional and suitable for smFRET assessment (A) Schematic representation of the domain structure of mouse KIF5A, including motor domain and coiled-coil (CC) regions 0–4. Key binding motifs are illustrated in green and unstructured regions in pink. (B) AlphaFold prediction of dimeric KIF5A, colored as domain structure above. Regions 1–371 and 411–1027 were modeled separately to show the elongated form. (C) Domain schematics illustrating CLIP, SNAP, and HaloTag positions in respective constructs. (D) Example kymographs of KIF5B(1–560)-HaloTag and CLIP-KIF5A(1–573) from single-molecule walking experiments (left) and overlaid with tracking data in magenta (right). Horizontal scale bars, 5 μm; vertical scale bars, 5 s; arrow indicates direction of time. (E) Analyzed particles from experiments in (D) showed no significant difference in track speed (*n* = 401 and 414 particles for CLIP-KIF5A(1–573) and KIF5B(1–560)-HaloTag, respectively, *p* = 0.7 by Mann-Whitney U) or motile percentage (*n* = 71 and *n* = 66 microtubules for KIF5B(1–560)-HaloTag and CLIP-KIF5A(1–573), respectively). Data from three biological replicates. (F) Single-molecule walking experiments of KIF5B(1–560)-HaloTag compared to CLIP-KIF5A-SNAP show intact autoinhibition of the latter. Arrowheads highlight non-processive binding events. Horizontal scale bars, 10 μm; vertical scale bars, 5 s; arrow indicates direction of time. (G) Coomassie stained SDS-PAGE gel showing pull down and elution of CLIP-KIF5A-SNAP using the incorporated FLAG tag. (H) Mass photometry of eluate in (G) with a 310 kDa peak corresponding to the tagged KIF5A dimer (predicted molecule weight 318 kDa).

We chose to focus the majority of our analysis on KIF5A (Figures 2A and 2B). The core of the mammalian kinesin-1 motor is a homodimer of two heavy chains, either KIF5A, KIF5B, or KIF5C. As with many protein complexes, AlphaFold has been able to provide the first structural predictions of the full kinesin-1 homodimer (Figures 2B, S2A, and S2B). These largely match previous predictions for the motor domain, coiled-coil stalk, and intrinsically disordered regions.^44^^,^^45^ However, there is one notable difference: previous models suggested that the major hinge that brought the head and tail of kinesin together was between CC1 and CC2.^35^^,^^46^ This unstructured region is now predicted to form a retrograde loop (residues V565 to A579) that facilitates the interlocking of these coiled coils together. It is also the case that structural predictions of the kinesin-1 stalk have changed with different versions of AlphaFold. The extended conformation of the stalk that was produced by earlier versions of AlphaFold (see both Figure S2C in this study and Figure 2-figure supplement 1 from Tan et al.^18^ as examples) has now become more compact, with coiled coils stacked against each other to create a concertina structure (Figures S2D and S2E). This predicted structure of the stalk is experimentally unverified at this time and is only apparent when the whole kinesin stalk domain is modeled in the absence of motor domains, to mimic possible active conformations of kinesin (Figure 2B). The latest version, AlphaFold3, produces a similar structure when the full stalk and tail is modeled in the absence of KLCs (Figure S2E). Within the stalk, flexible regions between CC2/3 and CC3/4 define two new hinge regions, hinge 1 and 2 (residues K685-T689 and M815-G821, respectively, Figure 2B), providing potential for a large range of movement in the kinesin tail. It is notable that structural predictions with KLCs^34^ suggest that KLCs act to stabilize the position of CC4 relative to CC3, which would impact this flexibility, even though hinge 2 still lacks a defined structure based on predicted local distance difference test (pLDDT) scores.

Having established CLIP and SNAP tags as suitable for smFRET, we moved to applying this labeling strategy to kinesin-1 by positioning CLIP and SNAP at the N and C termini of KIF5A (CLIP-KIF5A-SNAP, Figure 2C). However, before proceeding to smFRET analysis, we wanted to ensure that tagging kinesin at both the N and C termini simultaneously does not interfere with KIF5A motility or autoinhibition. *In vitro* reconstitution using cell lysates as a source of kinesin protein is a well-established technique for assessing kinesin motility on isolated microtubules.^18^^,^^21^^,^^47^^,^^48^ Using single-molecule total internal reflection fluorescence microscopy (TIRFM) assays (Figure 2D), comparing the motility of the constitutively active truncation CLIP-KIF5A(1–573) to well-studied C-terminally tagged KIF5B(1–560)-HaloTag, showed no difference in track speed or motile percentage (Figure 2E). Autoinhibition of full-length kinesin is also conserved in this assay system, with many fewer events typically observed compared to constitutively active mutants.^18^ Similarly, in comparison to KIF5B(1–560)-HaloTag, we observed robust autoinhibition of CLIP-KIF5A-SNAP in TIRFM assays (Figure 2F). Labeled CLIP-KIF5A-SNAP can be observed interacting with the microtubules, including diffusive movements across the lattice (Figure 2F, arrowheads), indicative of autoinhibited motors.^49^^,^^50^ However, unidirectional processive motility was absent. We conclude that motility and autoinhibition are conserved in CLIP-KIF5A-SNAP.

As a neuron-specific isoform of kinesin-1, KIF5A is not expressed endogenously in our HEK293 expression system, so co-purification with untagged endogenous kinesin-1 subunits is unlikely. To confirm that no associations of this type were interfering with our analysis, we performed both co-immunoprecipitation (Figure S2F) and mass photometry (Figures 2G and 2H). When HEK293 cells were transfected with CLIP-KIF5A-SNAP (or CLIP-KIF5B-SNAP), we were never able to observe co-immunoprecipitation with endogenous KIF5B (Figure S2F). To carry out a mass photometry analysis of complexes, we took advantage of the C-terminal FLAG included in our CLIP-SNAP expression vector (pCLAP). Complexes were enriched on FLAG-trap beads and eluted with the FLAG peptide prior to analysis (Figure 2G). The largest molecular weight complex observed with this method had a molecular weight of ∼310 kDa, corresponding well to the predicted size of a homodimer of CLIP-KIF5A-SNAP (Figure 2H). A consistent association with kinesin light chains would be expected to produce a peak at ∼450 kDa, but no peak is evident in this region. Likewise, no monomers of KIF5A were observed, indicating the homogeneity of the preparation. Having now established our expression system, labeling and assay procedures, we undertook an smFRET analysis of kinesin-1.

### Relative labeling efficiencies of SNAP and CLIP give dimeric molecules a unique stoichiometry signature in smFRET

**Figure 3:**
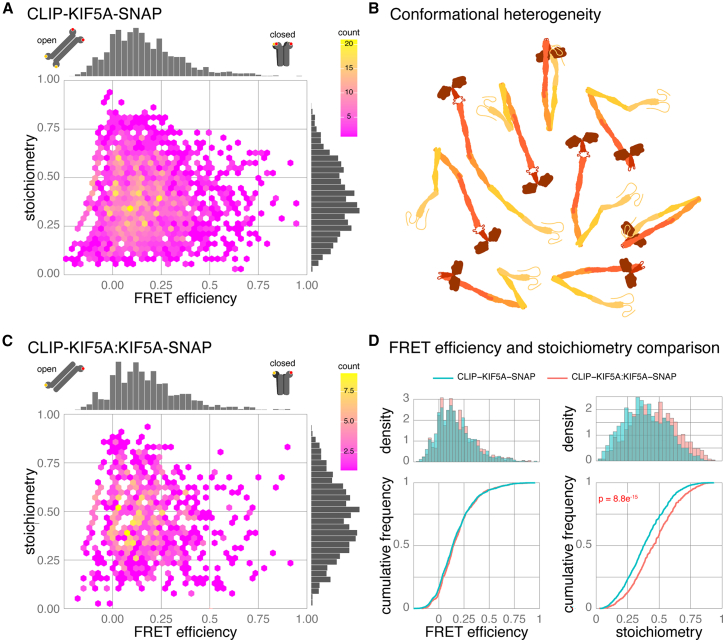
Tagging strategy affects stoichiometry but not FRET efficiency (A) Hexbin plot of FRET efficiency versus stoichiometry for CLIP-KIF5A-SNAP. Data combined from five biological replicates, *n* = 2,436 bursts. (B) Schematic representation of the conformational heterogeneity of kinesin-1. Each species is likely to have a very short lifetime. (C) Hexbin plot of FRET efficiency versus stoichiometry for heterodimers formed by CLIP-KIF5A:KIF5A-SNAP. Data from three biological replicates, *n* = 901 bursts. (D) Histograms and cumulative frequency plots of data from (A) and (C) comparing FRET efficiency and stoichiometry of KIF5A dimers formed of CLIP-KIF5A-SNAP (blue) or CLIP-KIF5A:KIF5A-SNAP (red).

Based on current models of kinesin-1 regulation, we anticipated that the majority of CLIP-KIF5A-SNAP should be in a closed conformation. It was also possible that we would observe both the open (low FRET) and closed (high FRET) conformations, as in recent negative-stain electron microscopy (EM) studies of purified kinesin.^17^^,^^18^ Plotting FRET efficiency versus stoichiometry for CLIP-KIF5A-SNAP FRET bursts shows a single continuous population with a long tail into the higher FRET regions of the axis (Figure 3A, median FRET efficiency is 0.136). The distribution of the histogram gives the relative abundance of conformations in a population. The distribution of CLIP-KIF5A-SNAP does not support a model of rapid transition between two distinct and stable conformational states, which would appear as two peaks. Instead, it suggests a rapidly interconverting population sampling a large conformational space (Figure 3B).

When we compared data on CLIP-KIF5A-SNAP to the CLIP-SNAP control protein, we also noticed a drop in stoichiometry; CLIP-SNAP stoichiometry is 0.49 ± 0.12, while CLIP-KIF5A-SNAP is 0.39 ± 0.17 (mean ± SD). Given that labeling kinetics are slower for CLIP-tag compared to SNAP-tag,^43^ this shift is most likely to have arisen from the difference in labeling efficiency between SNAP and CLIP for dimeric kinesin-1 (Figure S3A). This would result in a subpopulation of CLIP-KIF5A-SNAP dimers that carry two SNAP ligands for every CLIP ligand.

To test if labeling efficiency impacts stoichiometry and ensure this in turn does not affect our interpretation of the FRET efficiency distribution, we created KIF5A dimers, by co-transfection of KIF5A tagged with a single CLIP-tag or SNAP-tag (CLIP-KIF5A:KIF5A-SNAP). This strategy ensures that any dual-labeled molecules can only have one CLIP-tag and one SNAP-tag, ensuring equivalent labeling and a stoichiometry of 0.5. CLIP-KIF5A:KIF5A-SNAP dimers were labeled and analyzed with our optimized protocol. Plotting FRET efficiency versus stoichiometry of CLIP-KIF5A:KIF5A-SNAP shows the predicted increase in stoichiometry with mean ± SD of 0.46 ± 0.18 (Figures 3C and 3D). Furthermore, comparing the stoichiometry of biological replicates for the two labeling regimes highlights the consistency in stoichiometry for CLIP-KIF5A:KIF5A-SNAP compared to a more variable response for CLIP-KIF5A-SNAP dimers (Figures S3B and S3C). However, despite this impact on stoichiometry, the FRET efficiency distribution between the two labeling regimes was almost identical (Figure 3D), maintaining the skewed normal distribution with a long tail into the higher FRET regions of the axis.

One important side effect of the single-labeling approach is having to rely on the dimerization of CLIP-KIF5A:KIF5A-SNAP, accounting for (at most) 25% of the KIF5A dimers assembled. In practice, the percentage of dual-labeled kinesins is likely much lower for two reasons: first, not all cells are co-transfected, and second, data processing of smFRET photon bursts selects for molecules with both donor and acceptor fluorophore emission, losing molecules via inefficient CLIP labeling. Consequently, our ability to accumulate dual-labeled bursts from the single-tagging approach was severely impacted, greatly increasing the time for acquisition. A typical acquisition per biological replicate is three 15 min acquisition periods of the same sample, per construct (approximately an hour of instrument time); an FRET burst frequency at a quarter of the rate requires at least 4 h of acquisition. These long time periods at room temperature can potentially lead to sample integrity issues, particularly when multiple constructs are being compared within an experiment. Given this, combined with minimal impact on FRET efficiency distribution, we chose to use double-tagged kinesin constructs for our studies going forward.

### Changing the ionic environment alters the KIF5A conformational landscape

Given the long continuous FRET efficiency distribution we observed in our first experiments with tagged KIF5A (Figure 3), indicative of continuous sampling of many conformational states, we next sought to capture isolated populations of open or closed conformations.

To capture a closed conformation of KIF5A, we tried co-expressing CLIP-KIF5A(1–573) and KIF5A(574–1027)-SNAP. We reasoned that any co-labeled molecules we observed would have to be in the closed kinesin conformation, due to the interaction of the motor domains with the tail. However, we were never able to observe any dual-labeled FRET bursts with this approach. This is likely due to the low to moderate affinity K_D_ reported for the kinesin head and tail,^51^^,^^52^ making head-to-tail association highly unlikely in the pico-molar concentration range needed for successful smFRET experiments.

Altering the ionic strength of the assay buffer has been used for many years as a strategy for altering the conformation of kinesin-1. Original studies were able to indicate a transition from a compact state of kinesin to an elongated state by both rotary shadowed EM^26^ and changing sedimentation coefficients (a proxy for hydrodynamic radius),^27^^,^^51^ in response to increasing ionic strength up to an additional 1 M. Consequently, we predicted that the addition of NaCl to our assay buffer would alter the FRET efficiency distribution.

**Figure 4:**
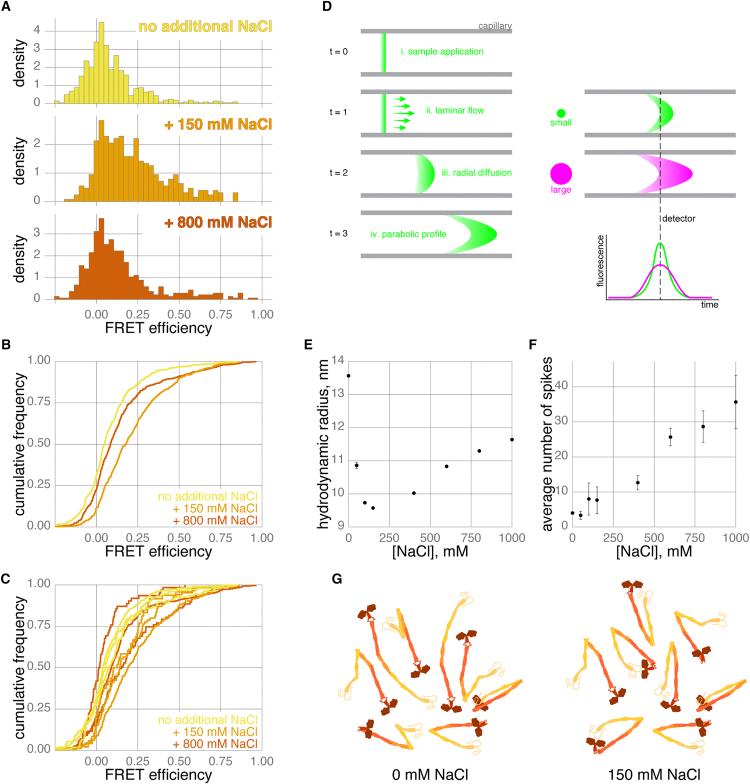
Changing salt concentration changes KIF5A behavior (A) Histograms of FRET efficiency for CLIP-KIF5A-SNAP in changing NaCl concentrations. Data combined from three biological replicates, *n* = 677, 421, and 449 bursts for 0 (yellow), 150 (orange), and 800 mM (brown) additional NaCl, respectively. (B) Cumulative frequency plots of data in (A) comparing FRET efficiency for CLIP-KIF5A-SNAP in changing NaCl concentrations. Pairwise Kolmogorov-Smirnov tests with Bonferroni correction gave the following *p* values: <2.2e^−16^ for no NaCl compared to +150 mM; 0.0037 for no NaCl compared to +800 mM NaCl; 2e^−8^ for +150 mM compared to +800 mM NaCl. (C) Cumulative frequency plots comparing FRET efficiency for individual biological replicates of CLIP-KIF5A-SNAP in changing NaCl concentrations. (D) Flow-induced dispersion analysis (FIDA) using a capillary. The sample is introduced to a thin capillary (i) and experiences laminar flow (ii), where fluid velocity is higher in the center compared to the walls of the capillary. Molecules also diffuse radially during this process (iii), which combined with laminar flow shapes the sample into a parabolic profile (iv). The fluorescence emitted by the molecules is acquired by a high sensitivity detection system and plotted against time (examples shown in Figure S4G). Molecule size determines radial diffusivity; small molecules (green) diffuse faster and create a more compact dispersion profile compared to large molecules (magenta). This allows for calculation of the hydrodynamic radius. (E) Apparent hydrodynamic radius of CLIP-KIF5A-SNAP for increasing additional NaCl concentrations determined by FIDA. Data points are mean ± standard deviation of three replicates. (F) Spikes, corresponding to aggregated protein, observed in analyzed Taylorgrams (see Figure S4G) for increasing additional NaCl concentrations. Data points are mean ± standard deviation of three replicates. (G) Schematic representation of the conformational heterogeneity of kinesin-1 in low versus 150 mM NaCl. In both cases, all species are likely to have a very short lifetime, but more compact conformations are more common in the presence of 150 mM NaCl.

To study the impact of increasing ionic strength on kinesin conformation, HEK293 cells were co-transfected with CLIP-KIF5A-SNAP and labeled with our optimized protocol. The same kinesin sample was then diluted either into smFRET assay buffer, which has low ionic strength, or assay buffer supplemented with an additional 150 or 800 mM NaCl, immediately prior to data collection. Accordingly, stoichiometry was consistent across samples (Figures S4A–S4C). However, changes in FRET efficiency were apparent. Compared to the no additional NaCl condition, adding 150 mM NaCl caused a significant increase in the FRET efficiency distribution, clearly visible in a shift to the right in the cumulative frequency distribution (Figures 4A and 4B). Adding 800 mM NaCl also caused an increase, but the shift was less pronounced. In fact, we found the response to 800 mM NaCl highly variable, often losing measurable kinesin molecules from the sample due to salting-out and precipitation, most obvious when comparing FRET efficiency across biological replicates (Figure 4C).

The increase in FRET efficiency at 150 mM NaCl represents an increase in the likelihood of the kinesin motor and tail being within 10 nm. This could be due to docking of the motor to the tail IAK motif, but raising the ionic concentration is more typically linked to the disruption of protein-protein interactions. It is possible that NaCl ions are screening charged patches on the kinesin stalk that normally create repulsion; for example, CC2 and CC3 have multiple surface-positive patches that may clash (Figure S4D). It could also arise from alteration in the conformation of hinges 1 and 2 (Figure 2B). For example, AlphaFold predicts (although it is experimentally unverified) hydrogen bonding and electrostatic interactions between the coiled coils of the kinesin-1 stalk that could be disrupted by increased NaCl (Figures S4E and S4F).

A large change in conformation from an elongated to a compact configuration should be observable as a change in the hydrodynamic radius (Rh) of kinesin, as Rh is the radius of a hypothetical sphere diffusing at the same rate as the molecule. To test this hypothesis, we performed FIDA (Figure 4D). FIDA directly measures diffusion of molecules undergoing lateral flow in a capillary and is highly sensitive to changes in Rh over a broad range of conditions in solution.^53^ FIDA is often used to measure K_D_, as size changes caused by ligand/protein binding are readily detectable.^54^ In contrast to confocal smFRET measurements, which are in the femto-molar range with single molecules observed for ∼2 ms, FIDA measurements are carried out in the nano-molar range, giving an ensemble measurement over a ∼2 min period. However, compared to other techniques that estimate Rh, such as size-exclusion chromatography or sucrose density centrifugation, FIDA is highly complementary to smFRET as measurements are direct, in solution, and on a much more comparable timescale (minutes rather than hours).

CLIP-KIF5A-SNAP was labeled with SNAP Alexa Fluor 488 ligand and prepared for analysis by binding and elution from FLAG beads (see STAR Methods). The Rh of KIF5A was then assessed by FIDA over a range of NaCl concentrations (Figures 4E, 4F, and S4G). In strong agreement to the smFRET measurements of kinesin in diluted cell lysate, FLAG-purified CLIP-KIF5A-SNAP was also in the most extended conformation in the absence of additional NaCl (mean Rh is 13.6 ± 0.03), reaching its most compact state with 150 mM NaCl (mean Rh is 9.57 ± 0.02). Similar to smFRET results, an extension at higher NaCl concentrations can also be observed (mean Rh at 800 mM is 11.30 ± 0.04), though this is still more compact than the conformation with no additional NaCl. Globular proteins demonstrate a proportional relationship between their molecular weight and their hydrodynamic radii, which becomes distorted by extended conformations. The molecular weight of a CLIP-KIF5A-SNAP dimer is ∼318 kDa, which if purely globular would give an Rh of 6.59 nm. However, the experimentally derived Rh of 13.6 nm corresponds to a molecular weight of 2.2 MDa for a globular protein, almost seven times the mass of a CLIP-KIF5A-SNAP dimer. Even at 150 mM NaCl, kinesin-1 is still relatively elongated, as an Rh of 9.57 nm corresponds to a molecular weight of ∼850 kDa for a globular protein. Another feature we noticed by FIDA was the increasing number of spikes in the signal with increasing NaCl concentration (Figures 4F and S4G), corresponding to protein aggregates passing through the capillary.^55^ This confirmed our observations from smFRET that kinesin-1 was likely salting out at high NaCl concentrations (Figure 4C).

Taken together, our results from FIDA and smFRET show excellent agreement overall, with the lowest FRET occurring in conditions with the highest Rh and vice versa. However, as smFRET does not average measurements over time or populations—a key advantage of single-molecule approaches—smFRET also reveals that even at 150 mM NaCl, kinesin-1 still exists as a conformational ensemble rather than a stable conformation (Figure 4G). Given the multiple conformations of kinesin-1 also observed by teams using size-exclusion chromatography at 150 mM NaCl,^17^^,^^18^^,^^33^ we think it most likely that the smFRET profile of CLIP-KIF5A-SNAP at 150 mM NaCl still represents a heterogeneous population with the equilibrium in this conformational landscape shifted to make the head-to-tail proximity more likely (Figure 4G).

### An activating mutation of KIF5A does not increase the frequency of the open conformation

**Figure 5:**
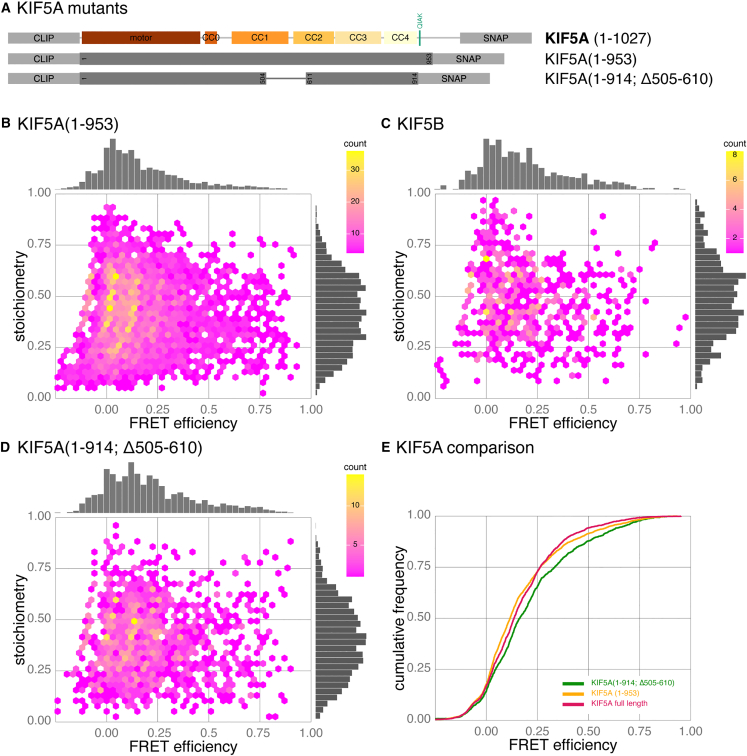
Activating mutations of KIF5A alter FRET efficiency (A) Schematic of KIF5A mutants used in relation to the domain structure of KIF5A. (B–D) Hexbin plots of FRET efficiency versus stoichiometry for CLIP-KIF5A(1–953)-SNAP (B), CLIP-KIF5B-SNAP (C), and CLIP-KIF5A(1–914; Δ505-610)-SNAP (D). Data combined from four to six biological replicates, *n* = 3,800, 666, and 1,475, bursts respectively. (E) Cumulative frequency plots of data in (B&D) comparing FRET efficiencies for KIF5A(1–953) and KIF5A(1–914; Δ505–610) mutants to full-length KIF5A (3A). Pairwise Kolmogorov-Smirnov tests with Bonferroni correction gave the following *p* values: 0.00052 for KIF5A compared to KIF5A(1–953); 7.6e^−6^ for KIF5A compared to KIF5A(1–914; Δ505–610); 2.6e^−12^ for KIF5A(1–953) compared to KIF5A(1–914; Δ505–610).

KIF5A has an extended disordered tail (Figure 2B) in comparison to KIF5B and KIF5C (106 compared to 40 and 32 residues, respectively), which could easily reduce any FRET efficiency measurements irrespective of the conformation of the coiled-coil stalk. To ensure this was not the case and that results are consistent across KIF5 isoforms, we analyzed a mutant KIF5A with the disordered region truncated to the same length as KIF5B. When analyzed by smFRET, CLIP-KIF5A(1–953)-SNAP shared the same key characteristics of the full-length KIF5A (Figures 5A, 5B, and 5E), namely one continuous skewed distribution that includes high-FRET events, but a median FRET efficiency of 0.116. As a direct comparison, we also performed smFRET analysis on CLIP-KIF5B-SNAP with similar results (Figure 5C, median FRET efficiency of 0.143). However, it was noticeable that KIF5B constructs were consistently more difficult to overexpress in HEK293 cells (Figure S5A), potentially due to intact degradation pathways and regulation of expression levels of an endogenous subunit, resulting in difficulty in accumulating bursts. When comparing the cumulative frequency plots of KIF5B to either full-length KIF5A or KIF5A(1–953), there was a subtle increase in FRET efficiency for KIF5B (Figures S5B and S5C, respectively); however, the overall distribution was broadly similar.

To further investigate the conformational landscape of kinesin-1, we created a double mutant that should retain much of the length of the motor while reducing autoinhibition (Figure 5A). In one of the key studies that revealed autoinhibition of kinesin-1, Friedman and Vale pinpointed residues 505–610 of human KIF5B as a potential hinge region facilitating the head-tail interaction of kinesin for autoinhibition. By deleting this region, both the velocity and event frequency of *in vitro* walking events was increased,^46^ an observation that was recently partially repeated although it does not activate the motor to the same extent as large tail truncations.^18^ The structure of this region, which includes the retrograde loop (highlighted in Figure S5D), is currently not predicted to be flexible by AlphaFold, but high-resolution structural data to confirm this is still lacking. In order to further open up the motor, we also deleted residues 915–1027 of the tail that includes the IAK motif, removing the head-tail interaction and creating a constitutively active motor.^18^ Consequently, we predicted that this mutant would show a decrease in FRET efficiency as it was far more likely to be in the open conformation. In fact, CLIP-KIF5A(1–914; Δ505–610)-SNAP showed an increase in FRET efficiency (Figures 5D and 5E; median FRET efficiency of 0.164). One possible explanation is that removing residues 505–610 (while retaining the coiled-coil structure through the heptad repeat, Figure S5E) disrupts predicted interactions across the stalk, increasing the likelihood of the head and tail coming into proximity despite the missing IAK motif. Alternatively, it may reflect that the conformational landscape is broadly the same between the wild type and the mutant but that the molecule is slightly shorter.

### Kinesin-1 is highly flexible, but crosslinking differentiates between activating kinesin mutants and intact autoinhibition

The long continuous distributions we observe by smFRET are suggestive of a broad conformational landscape and extensive flexibility in kinesin-1. To investigate the basis of this flexibility further, we calculated AlphaFold predictions of individual stalk domains (CC1-2, CC3, and CC4) in isolation across mammalian isoforms (Figure S6A). The predicted stalk-domain structures have high-confidence pLDDT scores for the majority of their length. However, it was noticeable that the phase of the predicted coiled-coil structures breaks down at the interface between these domains, accompanied by a decrease in pLDDT score (Figure S6A, gray boxes). This means that the phase of each individual coiled-coil domain is often out of sync from one coiled coil to the next, likely providing structural tension at the flexible regions of hinge 1 and hinge 2 (Figure 2B). Consequently, when CC1-4 is modeled as one complete stalk domain, the overall pLDDT confidence score drops, particularly around the hinges, a strong indicator of conformational flexibility in these regions (Figure S6B).

Based on the observed skewed, continuous, FRET efficiency distributions of kinesin-1, we hypothesize that kinesin-1 is sampling a large conformational space in solution, transiently bringing head and tail into closer proximity only some of the time. Recent negative-stain EM studies of kinesin-1 have made extensive use of crosslinking reagents to stabilize closed conformations after large-scale protein purification.^17^^,^^18^^,^^19^ In our kinesin preparations, we have not enriched for specific conformations by size-exclusion chromatography, maintaining a mixed heterogeneous population. However, we reasoned that crosslinking our full-length kinesin-1 would still capture some of these transient closed conformations, stabilizing them enough to decrease FRET efficiency.

To carry out crosslinking, labeled CLIP-KIF5A-SNAP was enriched from cell lysates with FLAG-trap beads, eluted with FLAG peptide and then subjected to crosslinking with a 1:1 ratio by mass with the amine-to-amine crosslinker bis(sulfosuccinimidyl)suberate, BS3. The BS3 ratio was determined to be optimal for preserving dimers without the over crosslinking observed at higher ratios (Figures S6C–S6E). We did observe an impact of crosslinking on stoichiometry within these experiments (Figure S6F; mean stoichiometry of 0.46 and 0.51 for uncrosslinked and crosslinked samples, respectively) likely due to stochastic disruption of the fluorophores by BS3. However, based on previous observations (Figure 3D), we do not think this negatively impacts our FRET efficiency observations (see the following paragraph).

**Figure 6:**
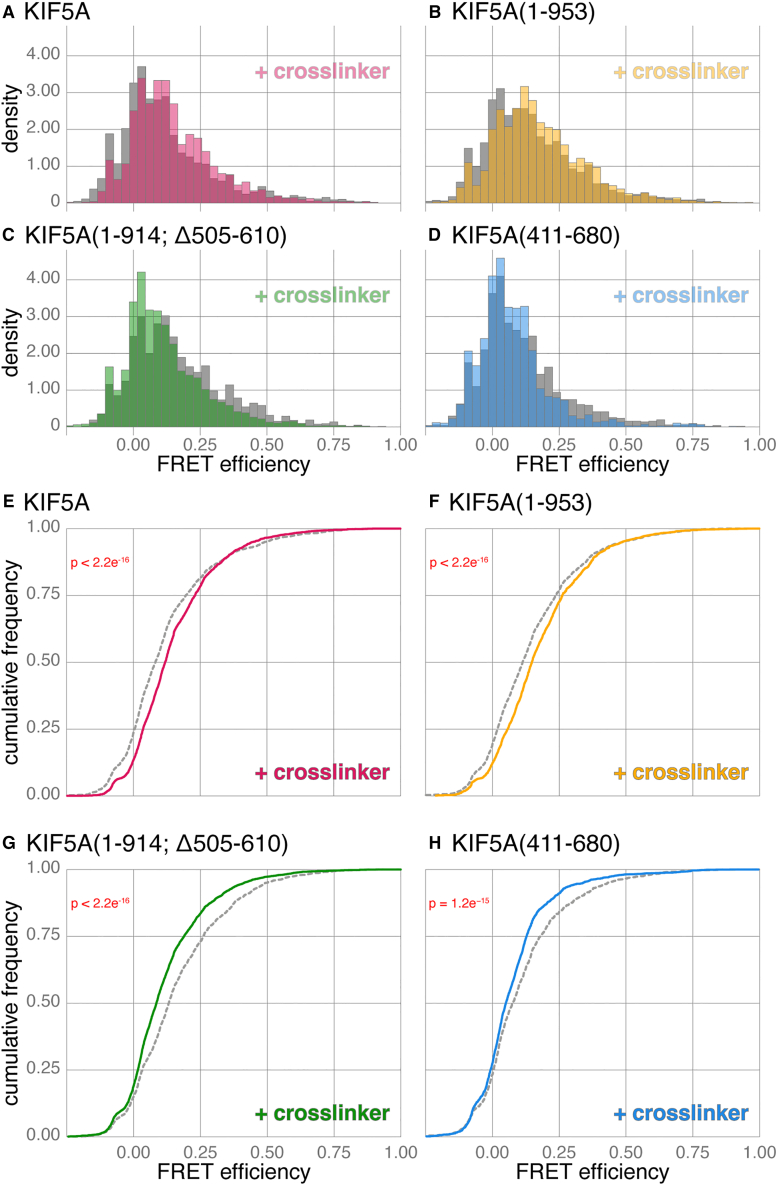
Crosslinking KIF5A constructs reveals a differential response to activating vs. truncating mutations (A–D) Histograms comparing the FRET efficiency of uncrosslinked (gray) and crosslinked (colored overlay) protein samples of KIF5A (A), KIF5A(1–953) (B), KIF5A(1–914; Δ505–610) (C), and KIF5A(411–680) (D). Data combined from five biological replicates, and *n* bursts for crosslinked and uncrosslinked samples as follows: KIF5A, 4093 and 3066; KIF5A(1–953), 4031 and 2873; KIF5A(1–914; Δ505–610), 6002 and 2626; and KIF5A(411–680), 3925 and 1700. (E−H) Cumulative frequency plots of data in (A)–(D) comparing FRET efficiencies of uncrosslinked (gray) and crosslinked (colored) protein samples of KIF5A (E), KIF5A(1–953) (F), KIF5A(1–914; Δ505–610) (G), and KIF5A(411–680) (H).

Compared to uncrosslinked kinesin from the same preparation, we observed a small but consistent increase in FRET efficiency for CLIP-KIF5A-SNAP (Figures 6A and 6E; median FRET efficiency for uncrosslinked and crosslinked is 0.082 and 0.119, respectively; a 45% increase), consistent with trapping some molecules with a head-tail interaction. A similar change is also observed for the shortened KIF5A that nevertheless preserves autoinhibition, CLIP-KIF5A(1–953)-SNAP (Figures 6B and 6F; median FRET efficiency for uncrosslinked and crosslinked is 0.113 and 0.147, respectively; a 30% increase). In further support of this model, CLIP-KIF5A(1–914; Δ505-610)-SNAP, the KIF5A construct where autoinhibition is disrupted, shows the opposite response to crosslinking with BS3, a decrease in FRET efficiency (Figures 6C and 6G; median FRET efficiency for uncrosslinked and crosslinked is 0.132 and 0.085, respectively; a 36% decrease), reflecting an increased tendency to occupy more open conformations.

AlphaFold-predicted structures of the combined CC1-2 domain consistently have the highest pLDDT scores of any domain within the kinesin-1 stalk, whether modeled alone or in combination with other domains (Figures S6A and S6B, see also Figure S7 for a historical overview of kinesin-1 domain naming conventions). To better understand whether this domain was contributing to the flexibility of the kinesin-1 motor, we carried out an smFRET analysis of CLIP-KIF5A(411–680)-SNAP with and without crosslinking. The CC1-2 domain is predicted by AlphaFold to be ∼35 nm long, and CLIP-KIF5A(411–680)-SNAP gave the lowest FRET efficiency of any construct we had observed to date (median FRET efficiency is 0.075), consistent with the majority of fluorophores being outside the range amenable to an FRET interaction (10 nm). However, despite this low-FRET efficiency, crosslinking CLIP-KIF5A(411–680)-SNAP was able to reduce the FRET efficiency even further (median FRET efficiency is 0.051, a 32% decrease). This result indicates that crosslinking CLIP-KIF5A(411–680)-SNAP stabilizes a more open conformation, suggesting that even CC1-2 retains some flexibility along its length and can therefore influence the conformational landscape of kinesin-1 (Figures 6D and 6H). Pairwise comparisons across this dataset further underscore the observed patterns (Figure S6G); although CLIP-KIF5A-SNAP and CLIP-KIF5A(1–953)-SNAP have similar profiles before and after crosslinking, CLIP-KIF5A-SNAP and CLIP-KIF5A(1–914; Δ505-610)-SNAP flip in relationship to one another, emphasizing the higher likelihood of the more active mutant being trapped in an open conformation.

### The presence of microtubules polarizes the kinesin-1 conformational landscape observed by smFRET

All smFRET and FIDA observations of kinesin-1 thus far in this study have been carried out in the absence of microtubules. However, first and foremost kinesin-1 is a microtubule-stimulated ATPase, and so its response to microtubules is an essential component for understanding how kinesin functions in an integrated system within cells.

**Figure 7:**
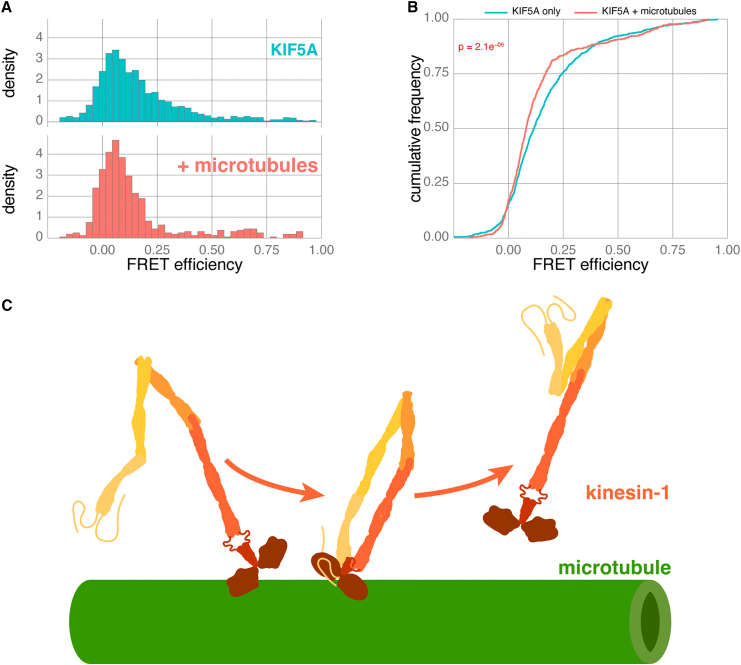
smFRET of KIF5A in the presence of microtubules reveals open and closed conformations (A) Histograms comparing the FRET efficiencies of KIF5A in the presence (red) and absence (blue) of microtubules. Data combined from 7 biological replicates, *n* = 529 and 1,247 bursts, respectively. (B) Cumulative frequency plots comparing FRET efficiencies of KIF5A in the presence and absence of microtubules. (C) Cartoon of kinesin inhibition model in the context of microtubules. Kinesin is autoinhibited in the context of microtubules when not itself bound to adapters and cargo but open to binding partners once released from the surface.

A key benefit of using confocal smFRET to study intramolecular kinesin dynamics is that kinesin conformation can be observed with and without microtubules or cargo. We therefore used smFRET to ask how the dynamic conformational landscape of kinesin motors is affected by microtubules. We added unlabeled, taxol-stabilized microtubules to our smFRET assay so that within the assay volume, kinesin-1 molecules were free to interact stochastically with the microtubules. However, unlike TIRF measurements, where kinesin observations are limited explicitly to those only interacting with the surface of the microtubule, confocal smFRET will always sample a mixed population of kinesins that may or may not be interacting with microtubules at the moment of observation. We reasoned that due to their larger size, kinesin bound to microtubules would diffuse more slowly through the confocal volume. We therefore compared photon bursts that were longer than 0.75 ms in duration, to improve our chances of capturing microtubule-interacting kinesins. Previous cryoelectron microscopy (cryo-EM) studies have been successful in trapping autoinhibited kinesin-1 motor domains bound to microtubules.^29^^,^^32^ Intriguingly, when we used smFRET to look at the distribution of kinesin molecules within our slower diffusing kinesin population, the FRET efficiency of CLIP-KIF5A-SNAP was more obviously polarized into two populations in the presence of microtubules compared to KIF5A alone (Figure 7A). For KIF5A with microtubules, the majority of FRET bursts have a low-FRET efficiency, indicative of an open conformation. There is also a slight increase in the abundance of a high-FRET population (FRET efficiency > 0.5), indicative of a closed conformation. Concomitant with this change, we observed a depletion of mid-FRET observations that previously created one continuous skewed population of KIF5A FRET efficiency (Figure 3A). The polarization into two distinct populations was also observable in the cumulative frequency plot (Figure 7B). At lower values of FRET efficiency, the cumulative frequency of KIF5A with microtubules (red) starts to the left of the KIF5A-only population (blue). However, because of the discontinuous distribution of KIF5A FRET in the presence of microtubules, these cumulative frequency plots cross over at higher FRET efficiency values. Despite the polarization we observe, there is no evidence in this experiment that the presence of microtubules is enough to stabilize a closed conformation; if this were the case, then the high-FRET population would be dominant in the histogram.

Based on our smFRET data in the presence of microtubules, we hypothesize that effective autoinhibition of the kinesin-1 homodimer is far more likely in the context of microtubules (Figure 7C). Indeed, the kinesin-1 tail has been known for some time to have microtubule-binding motifs,^56^ and these could effectively encourage the close apposition of the kinesin-1 head and tail in the context of the microtubule surface and the absence of cargo. This allows us to reconcile both the intact autoinhibition of kinesin-1 observed in TIRF assays (Figure 2F) and the observation that the majority of kinesin-1 is in an open conformation (Figures 3, 4, 5, and 6). Consequently, our model is that the closed state of kinesin-1 that promotes autoinhibition is very transient. It follows that, though transient, the closed state of full-length kinesin-1 (through an unknown mechanism) must promote a microtubule off-state, as otherwise kinesin-1 would decorate microtubules *in vitro*. We also conclude that the ability of kinesin-1 to be autoinhibited is not directly linked to the conformational changes observed by changing salt concentrations, as autoinhibition remains intact in low-salt conditions (Figure 2F). From a physiological perspective, it is essential that autoinhibition of kinesin in the absence of cargo should be resistant to changes in ionic strength over the physiological range; otherwise, kinesin would frequently become erroneously active.

## Discussion

We aimed to understand the structural changes that allow the kinesin-1 homodimer to transition between activated and autoinhibited states, by applying biophysical approaches that profile dynamic protein populations rather than static states. Contrary to expectations of a binary conformational landscape (open vs. closed), our findings reveal a broad spectrum of conformations adopted by kinesin-1 homodimers. Ionic strength plays a significant role in modulating this structural landscape. Based on both smFRET and FIDA experiments, we conclude that the majority of kinesin-1 homodimers in solution are in an open, rather than a closed, conformation. Interestingly, the presence of microtubules polarized this conformational landscape, potentially facilitating autoinhibition, while simultaneously promoting cargo search. It is important to note that the predominantly open conformation of kinesin-1 homodimers we observed here is not an impediment to effective autoinhibition, as this is still robust in TIRF microscopy microtubule walking assays using similar assay conditions. Consequently, we demonstrate that solution-based measurements can unlock valuable new information about the mechanisms underlying kinesin-1 homodimer function and regulation.

To bridge the gap between *in vitro* and *in vivo* studies of molecular machines, we developed and benchmarked a versatile expression and labeling approach in HEK293 cells. This approach has not previously been used for smFRET measurements, but we were inspired by the increasing popularity of *in vitro* motility assays using cell lysate as an important validation step in interpreting motor behavior within complex systems.^18^^,^^23^^,^^47^^,^^57^^,^^58^ All samples for smFRET were derived from a single 3.5 or 10 cm diameter well/dish using standard tissue culture procedures and commercially available cell-permeable fluorescent ligands. This approach enables the production of high-quality protein in sufficient quantities for both single-molecule measurements and downstream analyses like in-gel fluorescence. Using this method, we can easily obtain biological replicates and avoid relying on a single large-scale protein purification. Additionally, HEK293 cells are highly efficient at producing dimeric protein from one purification step with negligible degradation products (as demonstrated by mass photometry), eliminating the need for further purification steps. This approach was also readily able to produce protein for crosslinking and flash-freezing experiments, as well as labeling with brighter, cell-impermeable fluorophores, using one-step purification approaches. Beyond cost-effectiveness, this method opens up possibilities for studying protein responses to changes in the cellular environment in the future, such as kinase activation or alterations in gene expression. More importantly, we hope that by combining ready expression with an optimized labeling system and increasingly accessible technology platforms,^42^ it will lower the barrier to entry for biophysical characterization for many proteins and their complexes.

smFRET measurements of the distance between two fluorophores within a 3–10 nm range are highly reproducible. This is demonstrated within this study using our CLIP-SNAP fusion protein and in previous worldwide benchmark studies.^36^^,^^59^ The histogram of FRET efficiencies for CLIP-SNAP is relatively broad compared to other more rigid structures,^59^ because of the flexible linker between the domains. Conformational states of proteins are observable in smFRET, where there are discrete stable populations within the 10 nm observation window.^36^ However, we are yet to find discrete conformational states of kinesin-1 that are stable in solution. During this study, we found smFRET experiments much more replicable and robust than kinesin walking assays, where, although under-reported, performance can be highly variable from one day to the next. For TIRF-based walking assays, this variability has several sources; the quality of the surface preparation, microtubule preparation, motor preparation, and fluorescent labeling efficiency all play a significant role, in addition to the quality of the ATP and other reagents. By contrast, the smFRET assays are much less variable as there are far fewer critical variables in play.

Within our smFRET datasets, we have two fixed points that inform our interpretation of the data across the study. Our positive control CLIP-SNAP fusion protein joins the tags with a flexible linker (GSAGSAAGSGEF); when fully extended, this is ∼3.8 nm in length but is also capable of bringing the two tags within 1 nm of each other. Taking into account the position of the fluorophores in CLIP/SNAP from available crystal structures (6Y8P), the fluorophores could be positioned anywhere from 1 to 7.8 nm apart within the fusion protein control; because of the flexible nature of the linker, they are constantly sampling across this space, corresponding to the high-FRET efficiency (0.68) of this construct. Conversely, we observe a very low-FRET efficiency for KIF5A(411–680), which from structural predictions should form a relatively rigid coiled-coil structure approximately 35 nm long. If full-length KIF5A was in a stable open conformation, then we would expect a very similar FRET distribution histogram to KIF5A(411–680) both before and after crosslinking; although uncrosslinked samples have a similar smFRET profile, the crosslinked samples do not. If in a stable closed conformation, we would expect a histogram similar to the CLIP-SNAP fusion protein. If kinesin-1 was rapidly transitioning between two conformational states, we would anticipate a histogram with two distinct populations. However, we see none of these things but one long continuous distribution that samples both high-FRET states and low-FRET states, with the majority of the population sitting below 0.25 for all kinesin samples tested. Thus, our model is that kinesin-1 samples between both an open and a closed state as (1) the higher FRET efficiency events can only arise from a more closed conformational state and (2) this state cannot be stable, as otherwise it would be present as a distinct population in the histograms.

The interpretation of our results has been enhanced by combining both ensemble and single molecule in solution measurement techniques. Whereas smFRET accumulates information from observations of individual molecules ∼2 ms in duration, FIDA is based on the observation of a population ensemble ∼2 min in duration. By highlighting a decrease in the Rh, FIDA allows us to observe the large overall conformational change of kinesin-1 homodimers when the NaCl concentration is raised from 0 to 150 mM, while parallel smFRET tells us that this conformational change likely comes from closer association of the motor and tail of kinesin-1.

Soon after the discovery of kinesin-1,^60^^,^^61^ it was observed that changing ionic strength could produce large conformational changes. Hisanaga et al. used low-angle rotary shadowing EM and kinesin-1 purified from adrenal glands, to observe conformational diversity and a tendency to change from compact to elongated states in response to increasing ionic strength.^26^ Similar conformational changes were observed in ensemble measurements by sucrose gradient fractionation, using kinesin-1 purified from bovine brain^27^ and later recombinant drosophila KHC.^35^^,^^51^ The major difference between our own work and these original studies is the observation of an additional open conformation at low ionic strength, before closed conformations become more dominant at 150 mM NaCl. These differences are likely due to substantial differences in sample preparation strategies but also buffer composition and baseline ionic strength. On the other hand, we found it difficult to observe kinesin-1 in the presence of 800 mM NaCl by smFRET, likely because of the salting out effect and longer acquisition times needed for smFRET measurements. Salting out could readily be observed by the presence of an increasing number of aggregates by FIDA. Despite the limitations of carrying out single-molecule observations in high ionic strength, it is essential in order to facilitate comparison with biochemical studies of kinesin conformation,^26^^,^^27^^,^^35^^,^^51^ particularly given that these studies are the foundation of the binary (open/closed) model of kinesin-1 that has become synonymous with activation and autoinhibition. While open and closed states exist, our data point to the transient nature of these conformations in a highly flexible molecule that exists as a conformational ensemble.

During this investigation, we noticed that kinesin behavior is extremely sensitive not only to ionic concentration but also to buffering salts. The sensitivity of kinesin to its environment is also likely behind the very different size-exclusion chromatography profiles generated during kinesin-1 purification by different research teams.^17^^,^^18^^,^^33^ KIF5A in particular seems to have a stronger affinity for self-association under certain conditions^19^^,^^33^; however, we did not observe this within our own experimental procedures. Although a 150 mM NaCl environment is improbable within the cytosol, kinesin-1’s sensitivity to ionic strength hints at a potential response to physiological ion fluctuations, such as calcium, which was previously thought to be solely regulated by kinesin-interacting proteins like Miro1.^62^

The head-to-tail interaction of kinesin-1 was originally linked to its autoinhibition through both single-molecule motility^35^^,^^46^ and ATPase assays.^31^^,^^51^ This was subsequently validated by structural studies on isolated motor domains and peptides containing the conserved IAK motif from the tail.^29^^,^^30^ More recently, the dominant role of the IAK motif in preventing motility has been questioned. Mutating the IAK motif alone has little impact on enhancing the motility of kinesin-1 homodimers using *in vitro* motility assays.^18^^,^^33^ Similarly, cross-linking mass spectrometry of KIF5B and KIF5C did not reveal contacts between the IAK motif and kinesin motor domains, and the mutation of IAK was not sufficient to prevent the folding of kinesin-1.^18^ However, a similar cross-linking mass spectrometry study of KIF5A did find interactions between the motor domains and the IAK motif,^19^ and recent cryo-EM work with chimeric KIF5B molecules (consisting of only motor domains and IAK containing tail) does show the IAK docked between the motor domains when bound to microtubules.^32^ Given the large-scale conformational changes we have been able to observe here in the absence of microtubules, it seems highly unlikely that the IAK motif is solely responsible for opening and closing the kinesin motor, but it is still likely to have a direct role in the molecular mechanism of autoinhibition in response to microtubules.

AlphaFold predicts an interlocking of CC1 and CC2 to form a retrograde loop in the location previously ascribed to be a hinge in kinesin-1.^35^^,^^46^ In our experience, this prediction has been consistent across both AlphaFold versions and species (e.g., D. *melanogaster*, C. *elegans*, *Loligo pealeii*, and *Strongylocentrotus purpuratus*). Three recent low-resolution negative-stain EM studies captured the closed conformation of different isoforms of the full-length kinesin-1 motor.^17^^,^^18^^,^^19^ However, while providing valuable information, all studies used crosslinking to enrich the closed state and lack the high-resolution structural information to confirm the AlphaFold prediction of a more rigid (rather than hinge-like) CC1-CC2 region. *In vitro* motility and ATPase assays provided some of the original evidence that the region between CC1 and CC2 was the main region of flexibility mediating the head-to-tail interaction of kinesin-1.^35^^,^^46^ However, more recent results show a very limited impact on increasing motility when deleting this region, compared to constitutively active controls.^33^^,^^47^ Our data suggest that the Δ505-610 mutant is still highly flexible and likely capable of making head-to-tail interactions, having an FRET efficiency profile highly similar to full-length KIF5A. This is supported by previous results on a similar hinge mutant that formed a compact structure by the comparison of sedimentation coefficients.^35^ The overall flexibility of kinesin-1 along its length likely underpins the limited impact of mutating any one flexible region, hinge 1 or hinge 2, observed using *in vitro* motility assays.^18^ In support of a more rigid protein structure interlocking CC1 and CC2, we observed the lowest FRET efficiency in our study for the KIF5A(411–680) construct, a CC1-CC2 fragment. If residues 564–580 were a true hinge allowing large conformational changes in this region, we would expect a larger proportion of events to occupy higher FRET efficiency values. Consequently, we conclude that AlphaFold predictions are likely correct; however, it will be interesting to test if the interlocking coiled coils of CC1-CC2 can be separated by mutation or posttranslational modification in the future.

Although we did not study the impact of KLCs on the conformational landscape of kinesin-1 in this study, the two stalk hinges (hinge 1 and hinge 2) currently predicted by AlphaFold and presented here support the role of KLCs in promoting a closed conformation of kinesin-1. The KLC-binding site spans CC3 and CC4 and is thought to stabilize the region of flexibility (hinge 2) in between.^18^^,^^34^ It has been long established that the basal ATPase rate of kinesins with KLCs bound is lower than the heavy chains alone.^24^^,^^35^ Supporting evidence also points to a role of KLCs in favoring an autoinhibited conformation, in cells,^40^^,^^63^ through crosslinking studies^18^ and *in vitro* motility assays.^33^ However, both open and closed states of kinesin-1 heterotetramers (kinesin-1 additional light chains bound) can still be separated by size-exclusion chromatography,^17^^,^^18^^,^^34^ so it will be important to understand how the presence of KLCs alters the conformational landscape of kinesin-1 in the future.

Our work suggests that kinesin-1 does not have to be in a permanently closed conformation to have an effective autoinhibition mechanism. Kinesin is fundamentally a microtubule-stimulated ATPase^28^; if the time spent not bound to cargo and microtubules is insignificant, then ATPase activity away from the microtubule surface may be sufficiently low so as to not require further inhibition in the cellular context. From many *in vitro* studies to date, it is also clear that binding to microtubules is severely inhibited when kinesin is not bound to cargo.^21^^,^^22^^,^^33^ Consequently it is already safe to assume that cells tolerate the basal level of ATPase activity that kinesin is capable of when it is bound to cargo, but yet to find a microtubule. The other implication of our results is that binding to microtubules promotes the autoinhibited conformation when cargo is absent. Previous work proposed that the interaction of the kinesin tail with microtubules could support kinesin “pausing,”^29^ whereas it could have a more active role in autoinhibition. It will be interesting to investigate whether the previously identified ATP-independent microtubule-binding site preceding the IAK motif has a role in this inhibition.^31^^,^^56^^,^^63^^,^^64^

Kinesin-1 was discovered due to its role in fast axonal transport.^60^^,^^61^ Many studies since support the essential role of kinesin-1 in the maintenance of axons.^65^^,^^66^^,^^67^^,^^68^^,^^69^ In addition to fast transport, it is well established that many modes of slow axonal transport are also dependent on kinesin-1.^10^^,^^11^^,^^70^ Whereas fast axonal transport proceeds at close to the maximum speed of processive kinesins,^71^ slow transport is ∼10- to ∼100-fold slower.^72^ There is currently no biochemical or biophysical explanation for kinesin-1 activation by cargo that explains this discrepancy. We recently proposed that one possible mechanism for slow axonal transport is a rate-limiting supply of kinesin for certain classes of cargo.^10^ This allows for short bursts of motility in a cellular environment where many low-affinity cargos compete for a few free motors. However, if kinesin primarily exists in a stable locked down state, it is difficult to envision how this could be the case; the same series of high-affinity interactions would be needed to unlock the motor, regardless of whether a cargo is needed to stably recruit a motor or only to hang for a short burst. Due to the lack of high-resolution structural data, structural conformations of kinesin have often been inferred from *in vitro* and in cell activity assays. This could have masked the intrinsic conformational heterogeneity of kinesin-1, but the dynamics of the conformational ensembles are likely to be essential to kinesin-1 function in cells. A conformational ensemble, where kinesin-1 is constantly shifting between diverse open and closed states, allows for much greater diversity of cargo interactions, both stable recruitment and a more transient but promiscuous mode depending on the cellular environment.

Our hypothesis moving forward is that cargo recognition is about stabilizing the open conformation, rather than unfolding and “activating” kinesin, at least where KLC-independent functions of kinesin-1 are concerned. Further, preventing a head-to-tail interaction would be sufficient to promote a basal level of active transport. With this in mind, more possible scenarios of cargo recruitment are possible that could be better suited to the diverse array of cellular transport functions attributed to kinesin-1. With two key regions of flexibility in the stalk, hinge 1 and 2, the activity of kinesin-1 could be promoted through either (1) stabilizing hinge 1 and 2 into a fully elongated structure or (2) stabilizing the interaction of CC4 with CC3. In support of the first model, we previously predicted stabilization of an additional point of flexibility in the kinesin-1 homodimer, based on the activation of KIF5C by cooperation between the adapters HAP1 and GRIP1.^21^ We believe that by accounting for the intrinsic dynamics of kinesin dimers, solution-based measurements to study kinesin-1 conformational ensembles will be essential to building a comprehensive picture of both autoinhibition and cargo binding.

### Limitations of the study

There are several limitations to this study. Firstly, all observations are *in vitro*, often at very dilute concentrations; although cell lysate is present in many experiments, the concentration is far below that inside the cell. This means that molecular crowding might have an impact on the conformational landscape we can observe. Secondly, our smFRET measurements rely on N- and C-terminal tags. The dynamic range of smFRET is typically in the 3–10 nm range. Given the length of kinesin-1 at full extension is around 80 nm, the tag positions are beyond the detection limit of smFRET for fully open kinesin. Future studies will seek to position fluorophores internally with alternative labeling strategies. Finally, we have not studied the impact of well-known interaction partners, such as kinesin light chains or MAP7. Given that these proteins are key regulators of kinesin function, it will be important to understand how they impact the conformational landscape of kinesin-1.

## Resource availability

### Lead contact

All outputs are available as indicated. Further information and requests for resources and reagents should be directed to and will be fulfilled by the lead contact, Alison E. Twelvetrees (a.twelvetrees@sheffield.ac.uk).

### Materials availability

Plasmids generated in this study have been deposited to Addgene (https://www.addgene.org/browse/article/28252600/).

### Data and code availability

• All data have been deposited at Figshare and are publicly available as of the date of publication. Accession numbers are listed in the key resources table.
• All original analysis code has been deposited at Figshare and is publicly as of the date of publication. Accession numbers are listed in the key resources table.
• Example analysis code for processing raw smFRET data has been deposited at Figshare and is publicly as of the date of publication. Accession numbers are listed in the key resources table.

## Acknowledgments

We thank Dominic Bingham, Annie Savage, and Rebecca Mighell for generating some of the plasmids used in this study. We also thank Benjamin Ambrose and Elliot M. Steele for support conducting smFRET experiments. We are particularly indebted to Daniel Bose for constant discussion of the concepts underpinning the work and constructive comments on the manuscript. We would also like to thank the teams at FIDABio and Refeyn for their help in collecting and analyzing the FIDA and mass photometry data for this work, as well as the Wolfson Light Microscopy Facility and the Flow Cytometry Facility at the University of Sheffield.

This research was supported by the following funding: A.E.T. is a Sir Henry Dale Fellow, funded by the 10.13039/100010269Wellcome Trust and the 10.13039/501100000288Royal Society (grant number 220192/Z/20/Z); E.R.S. was supported by a studentship from the White Rose 10.13039/501100000268BBSRC Doctoral Training Partnership (grant number 2109768); E.D.T. was supported by a 10.13039/501100000858University of Sheffield PGT to PGR Scholarship from the Faculty of 10.13039/100018696Health. E.R.S. also received a 10.13039/501100000858University of Sheffield Postgraduate Research Student Publication Scholarship. The N-STORM microscope in the 10.13039/501100021076Wolfson Light Microscopy Facility was funded by 10.13039/501100000265MRC grant MK/K0157531/1.

## Author contributions

Conceptualization, A.E.T., T.D.C., and E.R.S.; data curation, E.R.S. and A.E.T.; formal analysis, E.R.S. and A.E.T.; funding acquisition, A.E.T., T.D.C., and E.R.S.; investigation, E.R.S. and E.D.T.; methodology, A.E.T., T.D.C., and E.R.S.; project administration, A.E.T.; supervision, A.E.T. and T.D.C.; resources, A.E.T. and T.D.C.; validation, E.R.S., A.E.T., and M.A.S.A.; visualization, A.E.T.; writing – original draft, A.E.T.; writing – review and editing, all authors.

## Declaration of interests

T.D.C. is the founder and CEO of Exciting Instruments (EI), a company that develops and sells instrumentation for single-molecule fluorescence experiments, including smFRET. M.A.S.A. is currently employed full time by EI. A.E.T. and E.R.S. held an EPSRC IAA grant in collaboration with EI that is unrelated to the work presented.

## STAR★Methods

### Key resources table

**Table:** 

REAGENT or RESOURCE	SOURCE	IDENTIFIER
**Antibodies**		

Alexa Fluor 680-AffiniPure Donkey Anti-Rabbit IgG (H + L)	Jackson ImmunoResearch Laboratories, Inc.	Cat#711-625-152; RRID:AB_2340627
Alexa Fluor 790-AffiniPure Donkey Anti-Mouse IgG (H + L)	Jackson ImmunoResearch Laboratories, Inc.	Cat#715-655-150; RRID:AB_2340870
Mouse anti-GAPDH	Proteintech	Cat#60004-1-1g; RRID:AB_2107436
Mouse anti-β-Tubulin antibody	Sigma-Aldrich	Cat#T5201-100UL; RRID:AB_609915
Rabbit anti-DYKDDDDK	Proteintech	Cat#20543-1-AP; RRID:AB_11232216
Rabbit anti-KIF5B	Proteintech	Cat#21632-1-AP; RRID:AB_11182931

**Chemicals, peptides, and recombinant proteins**		

3xDYKDDDDK-peptide	Proteintech	Cat#fp-1
BS3 (bis(sulfosuccinimidyl)suberate)	ThermoFisher Scientific	Cat#16091992
BSA	Sigma-Aldrich	Cat#AO281
CLIP-Cell TMR-Star	New England Biolabs	Cat#S9219S
DYKDDDDK Fab-Trap™ Agarose	Proteintech	Cat#ffa-20
Dynabead+ProtG	ThermoFisher Scientific	Cat#10003D
Fluorescent tubulin (HiLyte488)	Cytoskeleton	Cat#TL488M
HaloTag TMR Ligand	Promega	Cat#G8251
Paclitaxel	Cambridge Bioscience	Cat#CAY10461
Porcine brain tubulin	Cytoskeleton	Cat#T240
SNAP-Cell 647-SiR	New England Biolabs	Cat#S9102S
SNAP-Surface Alexa Fluor 488	New England Biolabs	Cat#S9129S
SNAP-Cell TMR-Star	New England Biolabs	Cat#S9105S
Dulbecco’s Modified Eagle Medium (DMEM) - high glucose	Sigma-Aldrich	Cat#D5796
Fetal Bovine Serum (FBS)	Gibco	Cat#10500-064
Penicillin Streptomycin Solution, 50X	Sigma-Aldrich	Cat#P0781
GlutaMAX, 100X	Gibco	Cat#35050038
Geneticin	Gibco	Cat#10131027
Sodium Pyruvate	Gibco	Cat#11360070
MEM Non-Essential Amino Acid Solution, 100X	Gibco	Cat#11140050
Trypsin-EDTA 0.5% Solution, 10X	Santa Cruz Biotechnology	Cat#sc-363354
Quick Coomassie Stain	Protein Ark	Cat#GEN-QC-STAIN-1L
LipoD293 DNA *In Vitro* Transfection Reagent	SignaGen Laboratories	Cat#SL100668
Pluronic F-127	Sigma-Aldrich	Cat#P2443-250G
Adenosine 5′-triphosphate (ATP) disodium salt hydrate	Sigma-Aldrich	Cat#A2383-1G
Glucose oxidase	Scientific Laboratory Supplies	Cat#G2133-50KU
Catalase	Sigma-Aldrich	Cat#C3155-50 MG

**Deposited data**		

All data	This paper	https://doi.org/10.15131/shef.data.c.7555284
All code	This paper	https://doi.org/10.15131/shef.data.c.7555284

**Experimental models: Cell lines**		

HEK293 cells line	ECACC	Cat#85120602; RRID:CVCL_0045
293FT cell line	ThermoFisher Scientific	Cat#R70007

**Recombinant DNA**		

pCLAP (pCDNA3.1-CLIP-SNAP-HisTag-FLAG)	This paper	RRID:Addgene_200786
pCDNA3.1-CLIP	This paper	RRID:Addgene_200787
pCDNA3.1-SNAP	This paper	RRID:Addgene_200788
pCDN3.1-StrepTag-HaloTag	This paper	RRID:Addgene_219682
CLIP-KIF5A-SNAP-HisTag-FLAG (pCLAP-KIF5A)	This paper	RRID:Addgene_200789
pCMV-KIF5B(1–560)-HaloTag-StrepTag (K560-Halo)	This paper	RRID:Addgene_219681
pCDNA3.1-CLIP-KIF5A(1–573)	This paper	RRID:Addgene_219683
pCDNA3.1-CLIP-KIF5A	This paper	RRID:Addgene_200790
pCDNA3.1-KIF5A-SNAP-HisTag-FLAG	This paper	RRID:Addgene_200791
pCDNA3.1-CLIP-KIF5A(Δ954-1027)-SNAP-HisTag-FLAG (pCLAP-KIF5A(Δ954-1027))	This paper	RRID:Addgene_200792
pCDNA3.1-CLIP-KIF5A(1–915;Δ505-610)-SNAP-HisTag-FLAG (pCLAP-KIF5A(1–915;Δ505-610))	This paper	RRID:Addgene_200793
pCDNA3.1-CLIP-KIF5A(411–680)-SNAP-HisTag-FLAG (pCLAP-KIF5A(411–680))	This paper	RRID:Addgene_200798
pCDNA3.1-CLIP-KIF5B-SNAP-HisTag-FLAG (pCLAP-KIF5B)	This paper	RRID:Addgene_200799

**Software and algorithms**		

Jupyter Notebook	https://jupyter.org/	–
FRETBursts python package	https://fretbursts.readthedocs.io/en/latest/	–
RStudio	https://cran.r-project.org/	–
DiscoverMP	https://www.refeyn.com/	–
Fida software 3.0	https://www.fidabio.com/	–
Fiji/ImageJ	https://imagej.net/software/fiji/	–
TrackMate v7.11.1	Ershov et al.^73^	–
AlphaFold v2.3.2 (unless otherwise stated)	https://colab.research.google.com/github/sokrypton/ColabFold/blob/main/AlphaFold2.ipynb	–

**Other**		

BD FACS Melody	BD Biosciences	–
Odyssey Fc Imager	LI-COR	–
Custom built smfBox	Ambrose et al.^42^	–
TwoMP mass photometer	Refeyn	–
Fida *Neo*	FidaBio	–
Ti-Ns N-STORM	Nikon	–
Custom built single-molecule scanning TIRF/FRAP imaging system	Cairn research	–

### Experimental model details

#### Cell culture

HEK 293 (Human Embryo Kidney 293) cells, were cultured in Dulbecco’s Modified Eagle Medium (DMEM) supplemented with 10% (vol/vol) Fetal Bovine Serum (FBS), 500 U Penicillin and 500 μg Streptomycin. 293FT cells (a clonal derivative of the Human Embryo Kidney 293 cell line), were cultured in DMEM supplemented with 10% FBS, 2 mM GlutaMAX, 500 μg/mL Geneticin, 1 mM Sodium Pyruvate, 1 X MEM Non-Essential Amino Acid Solution, 500 U Penicillin and 500 μg Streptomycin. Both cell lines were grown at 37 °C with 5% CO2 and passaged every 2–3 days when ∼80% confluent by trypsinization and replating. Both cell lines were checked regularly for mycoplasma contamination. HEK 293 cells were obtained from ECACC and the 293FT cells from ThermoFisher Scientific. They have not been independently authenticated.

### Method details

#### Recombinant DNA expression plasmids

All plasmids used in the smFRET assays were based on a pCDNA3.1-CLIP-SNAP-HisTag-FLAG plasmid we designed and named pCLAP. The CLIP tag is derived from the SNAP tag, with only eight amino acid changes, meaning DNA sequences are highly similar. To use both tags in the same construct, we codon optimised CLIP and SNAP linked by the short flexible linker sequence G-S-A-G-S-A-A-G-S-G-E-F^74^ for human expression, such that stretches of identity between the two nucleotide sequences were no longer than nine base pairs. Kinesin-1 (mouse KIF5A or human KIF5B) was inserted between the CLIP and SNAP tags creating CLIP-KIF5A-SNAP and CLIP-KIF5B-SNAP. Kinesin truncation mutants or single tagged constructs were generated by deletion mutagenesis PCR from these plasmids. Similarly, CLIP and SNAP only plasmids were generated by deletion mutagenesis PCR of pCLAP. All constructs with the SNAP-tag retain the C-terminal His and FLAG tags for protein purification and immuno-assays. The constitutively active human KIF5B construct (K560), C-terminally tagged with Halo and Strep tags, was derived from the previously published K560-HaloTag^10^ through inframe insertion of the Strep-tag at the C-terminus. The StrepTag-HaloTag construct was codon optimised for human expression and is used to show non-specific labeling of the HaloTag (Figure S1H). All plasmids are available from Addgene (see materials availability).

#### Transfection of cell lines

18 h after plating at ∼50% confluency in either a 6 well plate or 10 cm dish, cells were transfected using the LipoD293 DNA *In Vitro* Transfection Reagent. For a 6 well plate, media was removed so that only 1 mL was covering the cells. A volume of DNA equal to 1 μg was gently mixed into 50 μL of DMEM whilst 3 μL of transfection reagent was mixed into 50 μL of DMEM before being added dropwise to the diluted DNA solution. The DNA mixture was left to incubate at room temperature for 10 min before adding to the well in a dropwise manner. 24 h after transfection, cells were washed with 1 mL Phosphate Buffered Saline (PBS) and lifted with 500 μL of 0.5% trypsin for ∼5 min 1 mL of media was added to neutralise the trypsin and cells were harvested by centrifugation at 500 x g for 4 min at 4 °C. The cell pellet was resuspended in 1 mL PBS before a further centrifugation at 500 x g at 4 °C. All volumes were increased by a factor of five for a 10 cm dish.

#### Flow cytometry of ligand washout conditions

Untransfected HEK 293 cells were plated at ∼50% confluency into a six well plate. 24 h after plating, CLIP-Cell TMR-Star or SNAP-Cell 647-SiR was added to the cells at a final concentration of 0.25 μM and incubated for 1 h. The cells were washed three times with media and either harvested immediately or left to incubate for a further 1 to 4 h. Cells were harvested (as described above) and the pellet resuspended in 0.5 mL PBS. The cell suspension was analyzed using a BD FACS Melody flow cytometer (CLIP-Cell TMR-Star - 561 nm laser, PE 582/15 filter or SNAP-Cell 647-SiR - 633 nm laser, APC 660/10 filter).

#### In-gel fluorescence of ligand labeling

HEK 293 cells were plated into 6-well plates and transfected with either CLIP or SNAP (as described above). To optimise the dye incubation time, 24 h after transfection CLIP-Cell TMR-Star or SNAP-Cell 647-SiR was added to the cells for five different incubation periods (15 min, 30 min, 1 h, 2 h and overnight) at a final concentration of 0.25 μM. To optimise the dye concentration, 24 h after transfection CLIP-Cell TMR-Star or SNAP-Cell 647-SiR was added to cells at five different concentrations (0.1 μM, 0.25 μM, 0.5 μM, 1 μM and 2 μM) and left to incubate according to the previously optimised incubation conditions (overnight for CLIP-Cell TMR-Star and 2 h for SNAP-Cell 647-SiR). To determine the cross-reactivity between the two dyes, 24 h after transfection 0.25 μM of CLIP-Cell TMR-Star and SNAP-Cell 647-SiR were added to the cells and left to incubate either for 2 h or overnight. For all these experiments, cells were harvested (as described above) and lysed in 100 μL of lysis buffer (40 mM HEPES pH 7.5, 1 mM EDTA pH 8, 120 mM NaCl, 0.05% Triton X-100, 1 μg/mL Aprotinin, 10 μg/mL Leupeptin, 1 μg/mL Pepstatin A and 10 μg/mL tosyl-L-Arginine Methyl Ester) before incubation on ice for 10 min. Lysates were clarified by centrifugation at 17,000 x g for 10 min at 4 °C. 3X sample buffer (150 mM Tris pH 6.8, 6% SDS, 0.3 M DTT, 0.3% Bromophenol and 30% glycerol) was added to the resultant supernatant to a final 1X concentration. 20 μL of each sample was resolved by SDS-PAGE on 15% polyacrylamide resolving gels at 150V for ∼2 h. In-gel fluorescence of bound ligands was captured using the Odyssey Fc Imager (LI-COR) in the 600 nm and 700 nm channels.

#### Immunoprecipitation and western blotting

HEK 293 cells were plated into 6-well plates and transfected as described above. 24 h after transfection, media was removed and the cells scraped into 1.5 mL PBS. Cells were pelleted by centrifugation at 400 x g for 2 min at room temperature. The cell pellet was washed by resuspening in 1 mL PBS and centrifugation at 2300 x g for 5 min at 4 °C. Cells were lysed by resuspending the pellet in 300 μL of lysis buffer (50 mM HEPES pH 7.5, 1 mM EDTA pH 8, 1 mM MgCl_2_, 25 mM NaCl, 0.5% Triton X-100, 1 mM DTT, 1 μg/mL Aprotinin, 10 μg/mL Leupeptin, 1 μg/mL Pepstatin A and 10 μg/mL tosyl-L-Arginine Methyl Ester) and incubating on ice for 10 min. Lysates were clarified by centrifugation at 17,000 x g for 10 min at 4 °C. 1 μg of rabbit anti-DYKDDDDK was added to the supernatant and incubated with rotation for an hour at 4 °C. 25 μL of Dynabead+ProtG beads were washed three times by addition of 200 μL of PBS +0.02% Tween and discarding the supernatant. The cell lysate antibody mix was added to the beads and incubated with rotation for 10 min at room temperature. The supernatant was removed and the beads washed three times with 200 μL of PBS before resuspending in 1X sample buffer (50 mM Tris pH 6.8, 2% SDS, 0.1 M DTT, 0.1% Bromophenol and 10% glycerol). 20 μL of each sample was resolved by SDS-PAGE on a 10% polyacrylamide resolving gel and transferred to Immobilon-FL PVDF membrane. Membranes were probed with primary antibodies rabbit anti-DYKDDDDK, rabbit anti-KIF5B or mouse anti-GAPDH as indicated; fluorescent secondaries were Alexa Fluor 680-AffiniPure Donkey Anti-Rabbit or Alexa Fluor 790-AffiniPure Donkey Anti-Mouse. Fluorescent western membranes were visualised on the Odyssey Fc Imager (LI-COR).

#### smFRET assays

Six hours after transfection, CLIP-Cell TMR-Star was added to the HEK 293 cells in 6-well plates at a final concentration of 0.5 μM and left to incubate for around 15 h. SNAP-Cell 647-SiR (added at the same final concentration) was added to the cells for a further 2 h. The cells were then washed three times with media and incubated in 3 mL of fresh media for 2 h to remove any unbound dye. Cells were harvested as above and lysed in 50 μL of lysis buffer (40 mM HEPES pH 7.5, 1 mM EDTA pH 8, 120 mM NaCl, 0.05% Triton X-100, 0.1 mg/mL photobleached BSA, 1 mM DTT, 1 mM Mg.ATP, 1 μg/mL Aprotinin, 10 μg/mL Leupeptin, 1 μg/mL Pepstatin A and 10 μg/mL tosyl-L-Arginine Methyl Ester) before incubation on ice for 10 min. Lysates were clarified by centrifugation at 17,000 x g for 10 min at 4 °C. The supernatant was diluted in smFRET assay buffer (12 mM PIPES pH 6.7, 1 mM EGTA pH 7, 2 mM MgCl_2_, 0.1 mg/mL photobleached BSA, 1 mM DTT and 1 mM Mg.ATP) to result in single molecule resolution (usually 1:100,000). The sample was applied either to a custom built smFRET confocal microscope (smfBox) described in detail previously,^42^ or an EI-FLEX with the same configuration. Briefly, the smfBox consists of two lasers (80 mW 515 nm and 100 mW 638 nm), two emission filters (571/72 and 678/41) and two avalanche photodiodes; for the donor and acceptor respectively. Data collection was typically in three 15 min periods per sample. All labeled samples used for smFRET were also checked using in-gel fluorescence of samples resolved by SDS-PAGE on a 10% polyacrylamide gel followed by visualisation on an Odyssey Fc Imager (LI-COR).

#### Small batch protein purification and cross-linking

24 h after transfection, 10 cm dishes of 293FT cells were harvested by removing the media and the cells scraped into 5 mL PBS. Cells were pelleted by centrifugation at 400 x g for 2 min at room temperature and washed by resuspening in 1 mL PBS. After centrifugation at 2300 x g for 5 min at 4 °C the resultant pellet was resuspended in 200 μL lysis buffer (50 mM HEPES pH 7.5, 1 mM EDTA pH 8, 1 mM MgCl_2_, 25 mM KAc, 0.5% Triton X-100, 1 mM DTT, 1 μg/mL Aprotinin, 10 μg/mL Leupeptin, 1 μg/mL Pepstatin A and 10 μg/mL tosyl-L-Arginine Methyl Ester). 25 μL of DYKDDDDK Fab-Trap Agarose beads were washed three times by addition of 500 μL dilution buffer (50 mM HEPES pH 7.5, 1 mM EGTA pH 7, 2 mM MgCl_2_ and 150 mM KAc), pelleting by centrifugation at 2500*g* for 5 min at 4 °C and discarding the supernatant. The lysate (diluted in 300 μL dilution buffer) was equilibrated with the washed beads by incubation with rotation for 1 h at 4°C. For on bead labeling, 2 μL of each ligand (CLIP-Cell TMR-Star and SNAP-Cell 647-SiR, 600 μM stock) was also added to the lysate. After incubation, the beads were washed three times by centrifugation at 2500 x g for 5 min at 4 °C and resuspension in 500 μL wash buffer (50 mM HEPES pH 7.5, 1 mM EDTA pH 8, 2 mM MgCl_2_, 150 mM KAc, 1 mM DTT, 1 μg/mL Aprotinin, 10 μg/mL Leupeptin, 1 μg/mL Pepstatin A and 10 μg/mL tosyl-L-Arginine Methyl Ester). 3xDYKDDDDK-peptide was diluted to 150 μg/mL with PBS and 100 μL added to the washed beads. This suspension was incubated with rotation for 20 min at room temperature. Eluted protein was separated from beads by centrifugation at 2500 x g for 2 min at 4 °C. Protein concentration was determined by BCA assay. If cross-linking was required, BS3 was diluted to 2 mg/mL in the dilution buffer and added to half the sample at a 1:1 mass ratio. The mixture was incubated on ice for 20 min, before cross-linking was quenched by addition of Tris pH 7.5 to a final concentration of 25 mM and further incubated on ice for 20 min. Gel samples of each stage were analyzed by in-gel fluorescence with 20 μL of each sample resolved by SDS-PAGE with a 10% polyacrylamide resolving gel. Following fluorescence imaging, the gel was stained with Quick Coomassie Stain (Protein Ark) and total protein imaged for comparison.

#### Mass photometry

Samples were prepared as above by small batch protein purification without addition of the crosslinker and analyzed using the Refeyn TwoMP mass photometer. Measurements were carried out at room temperature.

#### Flow induced dispersion analysis

Samples were prepared as above by small batch protein purification without addition of the crosslinker. To label the protein, 2 μL of 0.6 mM SNAP-Surface Alexa Fluor 488 was added to the lysate before addition to the beads. Protein was analyzed using a Fida *Neo* instrument with an excitation wavelength of 480 nm. Purified CLIP-KIF5A-SNAP was mixed with the corresponding BRB80 buffer (80 mM PIPES pH 6.7, 1 mM EGTA pH 7, 2 mM MgCl_2_) supplemented with the relevant NaCl concentration (0–1 M) within the instrument, immediately prior to measurement. Each salt concentration was run in triplicate.

#### Microtubule polymerisation for *in vitro* assays

Lyophilised tubulins (both unlabelled and HiLyte 488 labeled) were resuspended to 5 mg/mL in GBRB80 (1 mM GTP, 80 mM PIPES pH 6.7, 1 mM EGTA pH 7, 2 mM MgCl_2_), flash frozen and stored at −80°C. 40 μL of this tubulin stock (0.8:40 ratio of labeled:unlabelled tubulin if making fluorescent microtubules) was thawed on ice and centrifuged at 270,000 x g (TLA-100.3 rotor) for 10 min at 4 °C. The supernatant was then incubated at 37 °C for 20 min before addition of Paclitaxel to a final concentration of 40 μM and further incubation for 20 min. Microtubules were pelleted by centrifugation at 270,000 x g (TLA-100.3 rotor) for 10 min at room temperature and gently resuspended in TBRB80 (40 μM Paclitaxel, 80 mM PIPES pH 6.7, 1 mM EGTA pH 7, 2 mM MgCl_2_). Microtubules were kept in the dark at room temperature until use. Microtubules used in smFRET assays were sheared by passing through a Hamilton 25 μL 22G fixed needle syringe ten times before diluting 1 μL in 250 μL smFRET assay buffer (12 mM PIPES pH 6.7, 1 mM EGTA pH 7, 2 mM MgCl_2_, 0.1 mg/mL photobleached BSA, 1 mM DTT and 1 mM Mg.ATP) and using this to dilute the CLIP-KIF5A-SNAP transfected cell lysate.

#### Single molecule motility assays

24 h after transfection, HaloTag TMR, CLIP-Cell TMR-Star or SNAP-Cell TMR-Star ligands were added to 293FT cells. HaloTag TMR was added at a final concentration of 25 nM and incubated on the cells for 15 min. The cells were then washed three times with fresh media and incubated for a further 30 min. CLIP-Cell TMR-Star or SNAP-Cell TMR-Star were added and washed as above for smFRET assays. Cells were harvested and washed in PBS as stated above for immunoprecipitation. The cell pellet was resuspended in 300 μL of lysis buffer (50 mM Tris pH 8, 25 mM KAc, 1 mM MgCl_2_, 1 mM EDTA, 0.5% Triton X-100, 1 mM DTT, 1 μg/mL Aprotinin, 10 μg/mL Leupeptin, 1 μg/mL Pepstatin A and 10 μg/mL tosyl-L-Arginine Methyl Ester) and incubated on ice for 10 min before centrifugation at 16,000 x g for 10 min at 4 °C. The supernatant was collected and stored on ice for use in flow chambers (below).

Flow chambers were prepared as described previously.^21^ Briefly, a #1.5 22 × 32 mm cleaned and silanised coverslip was attached to a glass microscope slide using double-sided tape. Chambers were defined using vacuum grease. Flow cells were incubated for 5 min with i) 1 chamber volume (CV) of 20 μg/mL anti-β-Tubulin antibody (diluted in BRB80), ii) 1 CV of 50 mg/mL F-127, iii) 2 CV of microtubules diluted in TBRB80 (1:250). Unbound microtubules were removed with a 2 CV BRB80 wash step before adding the final reaction mix of 1 CV formed of 1 μL cell lysate in 20 μL of assay buffer (80 mM PIPES pH 6.7, 1 mM EGTA pH 7, 1 mM MgCl_2_, 0.3 mg/mL casein, 0.3 mg/mL BSA 10 mM DTT, 10 mM ATP, 15 mg/mL glucose, 0.5 μg/mL glucose oxidase and 470 U/ml catalase).

Motility assays containing CLIPf-KIF5A-SNAPf (Figure 2F) were imaged using the Nikon Ti-NS N-STORM microscope at the Wolfson Light Microscopy Facility with the SR Apo TIRF 100× objective lens and the Andor iXion ultra EM-CCD camera, with 488 nm and 561 nm lasers. Movies were acquired for 1 min at 40 ms exposure. KIF5B(1–560)-HaloTag and CLIPf−KIF5A(1–573) constructs (Figure 2D) were imaged on a custom single-molecule scanning TIRF/FRAP imaging system built by Cairn Research. This setup uses a Ti2-E automated inverted microscope base (Nikon), ASI automated XY & piezo-Z stage controller, iLas 2 scanning TIRF/FRAP unit (Gataca Systems) with dual-collimation optics, Cairn multi-line laserbank, and a Photometrics Prime 95B sCMOS camera. TIRF was performed with a 488 nm and 561 nm laser, a TIRF quad-band filter cube (TRF89901-EMv2, Chroma) and additional emission clean up provided by a Cairn Optospin filter wheel with ET525/50m or ET595/44m filters for 488 and 561 laser lines respectively. Images were taken in MetaMorph acquisition software using a Nikon CFI Apochromat TIRF 100XC oil (N.A. 1.49, W.D. 0.12 mm) objective lens. Exposure time was 40 ms (25 frames per second) and movies were acquired for 1000 frames.

#### Protein structure prediction

To predict three-dimensional structures of the target proteins, we used AlphaFold v2.3.2 (unless otherwise stated),^75^^,^^76^ using the AlphaFold Colab notebook hosted on Google Colaboratory.^77^ For all structures the relaxation stage was enabled and multimer model recycling was set to a limit of six. The highest scoring prediction was used. Structures and associated files are available from ORDA (see data and code availability).

### Quantification and statistical analysis

#### Flow cytometry of ligand washout conditions

The raw data was transformed and the fluorescence intensity plotted using a custom R notebook (see data and code availability) and the flowcore package.^78^ The data from each biological repeat was combined and the median value for each sample compared.

#### In-gel fluorescence of ligand labeling

The signal intensity for each band was normalised to the lowest incubation or concentration per biological repeat and plotted using R (see data and code availability, below).

#### smFRET assays

Data was analyzed using a custom built Jupyter (for initial elimination of background and burst selection using dye specific correction factors using the FRETBursts package^79^) and R (for burst filtration and plotting) notebooks (see data and code availability, below). The burst threshold was set at 20 photons in both the DD + DA and AA channels for the burst selection. Any technical repeats with a burst rate above 1 burst per second were eliminated and the background filters were set as above: 8.5 photons for donor excitation (DD + DA), 2.6 for donor emission (DD) and 5 for acceptor excitation and emission (AA). Displayed *p* values for paired comparisons were calculated by Kolmogorov-Smirnov test. For multiple comparisons, pairwise Kolmogorov-Smirnov tests were performed with Bonferroni correction.

#### Mass photometry

All resultant images were analyzed using the Refeyn DiscoverMP software and conversion from interferometric contrast to molecular weights was calibrated using Refeyn’s protein standards. Gaussian fitting to mass histograms was performed using R (see data and code availability, below).

#### Flow induced dispersion analysis

Taylorgrams were fitted using the standard parameters and smooth curve settings in the FIDA software (version 3.0) to determine hydrodynamic radii and spike count for each condition. This data was plotted using R (see data and code availability, below).

#### Hydrodynamic radius calculation

Estimates of hydrodynamic radius for a globular protein of known mass were calculated using FIDA software.

#### Single molecule motility assays

Motility in image stacks was analyzed using Fiji^80^ and the TrackMate (v7.11.1) plugin.^73^ Briefly, individual microtubules were isolated from the field of view using the line selection tool followed by the Straighten tool in Fiji. Kinesin molecules were tracked by running TrackMate on the 561 channel, using the LoG detector, an estimated object diameter of 8 pixels and the LAP tracker function. The detected spots were additionally filtered by their Y position (i.e., proximity to the straightened microtubule). Note that stationary puncta that were present for the duration of the capture were excluded from the analysis, as these cannot be differentiated from non-specifically bound species on the glass surface. Further downstream analysis was performed in R (see data and code availability).
