# Supplementary material for: Kinesin-1 is highly flexible and adopts an open conformation in the absence of cargo
Source: iScience. 2026 Feb 2;29(3):114875. doi: 10.1016/j.isci.2026.114875 (PMC12925526; doi:10.1016/j.isci.2026.114875)
Supplement: Document S1. Figures S1–S7 [file mmc1.pdf]

iScience, Volume *29*

Supplemental information

Kinesin-1 is highly flexible and adopts an open conformation in the absence of cargo

Evelyn R. Smith, Emma D. Turner, Mahmoud A.S. Abdelhamid, Timothy D. Craggs, and Alison E. Twelvetrees

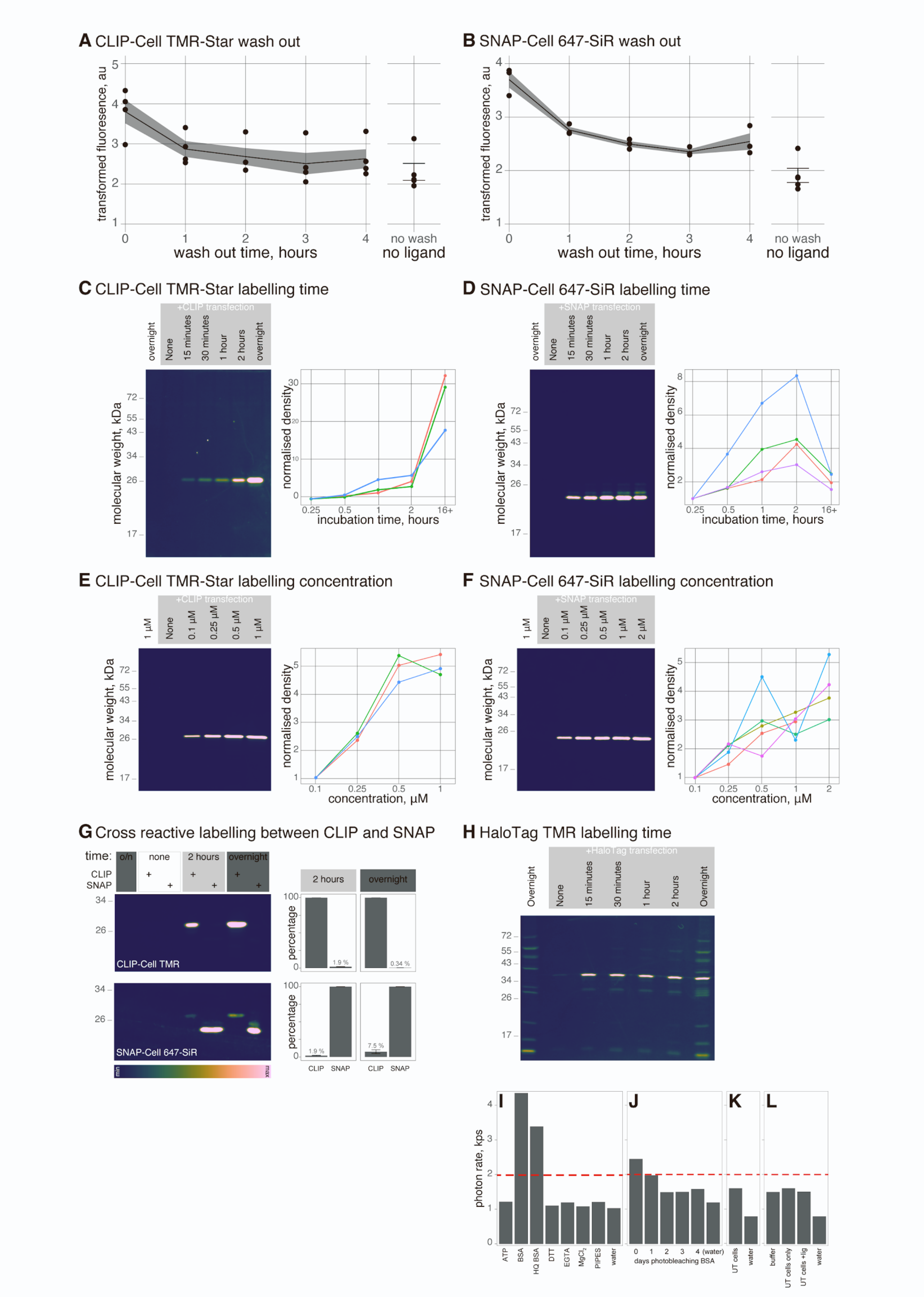

Figure S1. Optimising labelling of CLIP and SNAP in HEK 293 cells, related to Figure 1.
A, B. Flow cytometry results showing time taken to wash out unbound CLIP-Cell TMR-Star (A) and SNAP-Cell 647-SiR (B) from non-transfected HEK 293 cells. Fluorescence signal from untransfected and

unlabelled cells is shown for comparison. At least three biological replicates were acquired for each time point. Line indicates the median with grey shading showing the standard error.
C, D. In-gel fluorescence of cell lysate from HEK 293 cells transfected with CLIP or SNAP alone and incubated with CLIP-Cell TMR-Star (C) or SNAP-Cell 647-SiR (D) for the indicated time periods. Quantified signal intensity for biological replicates is shown, with labelling intensity normalised to the first time point. At least three biological replicates were acquired for each time point. The batlow scientific colour map was applied to all fluorescent gel images to indicate intensity, and is illustrated in panel G.
E, F. In-gel fluorescence of cell lysate from HEK 293 cells transfected with CLIP or SNAP alone and incubated with CLIP-Cell TMR-Star (E) or SNAP-Cell 647-SiR (F) at the indicated concentrations for 2 hours or overnight for SNAP and CLIP respectively. Quantified signal intensity for biological replicates is shown, with labelling intensity normalised to the first concentration point. At least three biological replicates were acquired for each concentration. The batlow scientific colour map was applied to all fluorescent gel images to indicate intensity, and is illustrated in panel G.
G. Cross reactivity of CLIP and SNAP ligands for their counter tag was measured by in-gel fluorescence of cell lysate from HEK 293 cells, transfected with either CLIP-tag or SNAP-tag and incubated with ligands for time periods as indicated. Even overnight labelling with CLIP-Cell TMR-Star produced minimal cross reactive labelling with the SNAP-tag (0.34%), however SNAP-Cell 647-SiR did have more significant cross reactivity with CLIP-tags over extended time periods (7.5%). Consequently CLIP-Cell TMR-Star was incubated on transfected HEK cells overnight, whilst SNAP-Cell 647-SiR was limited to 2 hours before washing and lysate collection.
H. In-gel fluorescence revealed significant non-specific labelling of the HaloTag TMR ligand when incubated with HEK 293 cells for longer than 30 minutes, both in HaloTag transfected (grey box) and untransfected cells. The batlow scientific colour map was applied to all fluorescent gel images to indicate intensity, and is illustrated in panel G.
I-L. Background photon rates (kilo photons per second, kps) observed in components of the smFRET assay buffer. Red dotted line marks the threshold for the photon rate, above which background photons begin to interfere with burst analysis. When each buffer component was assayed individually (I), BSA (even high quality BSA) was found to be a significant source of background photon bursts when illuminated by ALEX. Photobleaching the BSA stock over several days (J) in a lab-made broad wavelength illumination box, reduced this background to acceptable levels. Cell lysate from untransfected cells diluted 10,000 fold (comparable to typical assay conditions in smFRET) does not add considerably to the background when compared to water alone (K). In the optimised final smFRET assay conditions (L) the kinesin smFRET assay buffer (made with photo bleached BSA, buffer), untransfected HEK 293 cell lysate diluted in assay buffer (cells only), and diluted lysate from cells treated with both fluorophores and washed out according to the optimised labelling conditions (cells +lig), all displayed background photon rates low enough for reliable smFRET analysis. Data in L was collected routinely to ensure sample and buffer integrity prior to data collection.

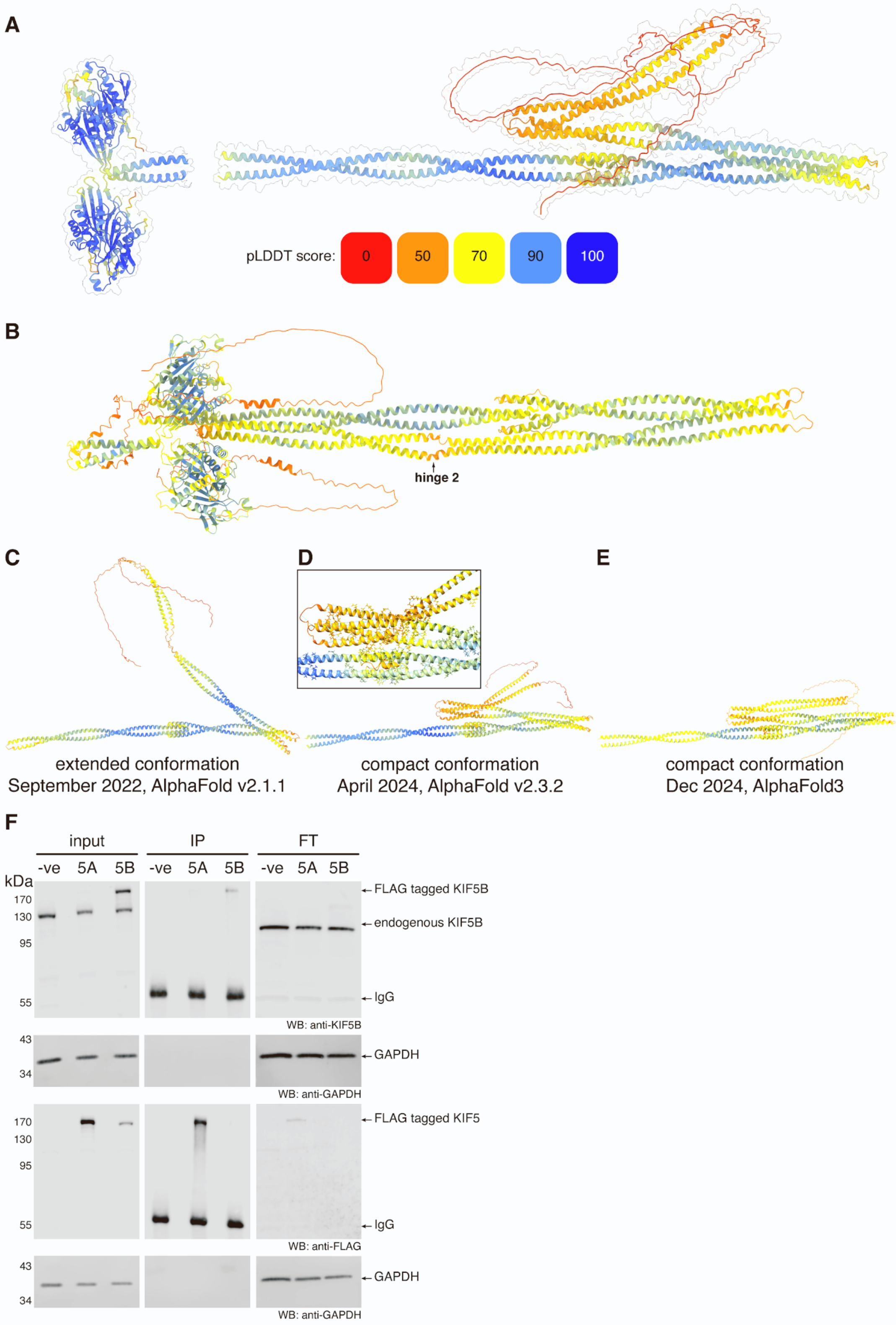

Figure S2. AlphaFold predictions of the kinesin-1 stalk have compacted over release versions, related to Figure 2.

A. The same AlphaFold2 model shown in Figure 2B, coloured by pLDDT score.

B. An AlphaFold3 model of full length KIF5A, coloured by pLDDT score and showing a potential closed conformation. Hinge 2 is still visible as a region of low confidence. Note that the site of IAK motif docking on the motor domains is not supported by previously published structural analysis [S1, S2].

C-E. AlphaFold structures of human KIF5C(413-957), encompassing the stalk and tail, generated in AlphaFold v2.1.1 (C), v2.3.2 (D), and v3 (E) coloured by pLDDT score. Inset in panel D highlights the

increased hydrogen bonding and electrostatic interactions driving compaction of coiled coils across CC1-CC4.

F. Exogenous KIF5A does not co-immunoprecipitate endogenous KIF5B from HEK 293 cells. Our CLIP-SNAP expression vector (pCLAP) also contains a C-terminal FLAG tag. HEK 293 cells were transfected with mouse CLIP-KIF5A-SNAP-FLAG (5A) or human CLIP-KIF5B-SNAP-FLAG (5B). The overexpressed proteins were immunoprecipitated with anti-FLAG antibody. Samples were separated by SDS-PAGE and probed by western blot (WB) using the antibodies: rabbit anti-KIF5B, mouse anti-GAPDH and rabbit anti-FLAG. Although KIF5A was readily immunoprecipitated, no corresponding endogenous KIF5B band was observed. Tagged KIF5B did not overexpress to the same level as KIF5A, but with the endogenous KIF5B antibody a similar result could be observed. Samples: input, cleared cell lysate; IP, immunoprecipitation; FT, flow through. Blots representative of three biological replicates.

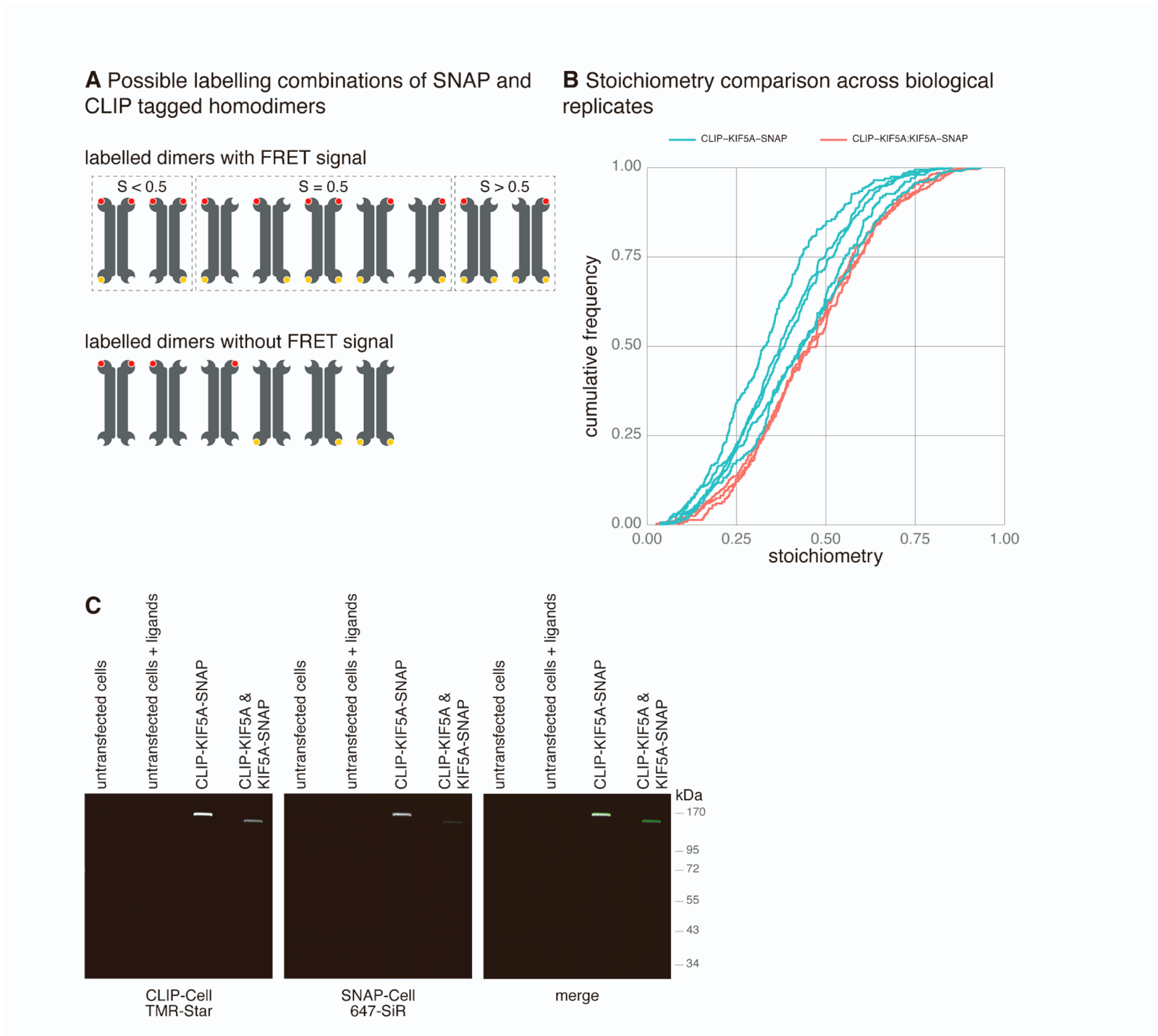

Figure S3. Variable stoichiometry across biological replicates for homodimers of CLIP-KIF5A-SNAP, related to Figure 3.

A. Schematic of possible labelling stoichiometries of CLIP-SNAP tagged homodimers and the corresponding stoichiometry value. Labelled CLIP and SNAP are yellow and red circles respectively.

B. Cumulative frequency plots of stoichiometry for individual biological replicates of CLIP-KIF5A-SNAP homodimers (blue) compared to CLIP-KIF5A & KIF5A-SNAP heterodimers (red). Homodimers show more variability in the stoichiometry of labelling, due to the variable labelling efficiency of CLIP compared to SNAP. The low proportion of observed heterodimer molecules poses challenges for collecting data in this regime, despite a more consistent stoichiometry from eliminating labelling variability.

C. In-gel fluorescence of cell lysate from HEK 293 cells transfected with CLIP-KIF5A-SNAP or CLIP-KIF5A & KIF5A-SNAP heterodimers, and incubated with CLIP-Cell TMR-Star and SNAP-Cell 647-SiR. The merged fluorescence panel (right) shows 647-SiR in magenta and TMR-Star in green. CLIP-KIF5A or KIF5A-SNAP are a lower molecular weight as they are shorter by the equivalent of one tag (~20 kDa) compared to CLIP-KIF5A-SNAP.

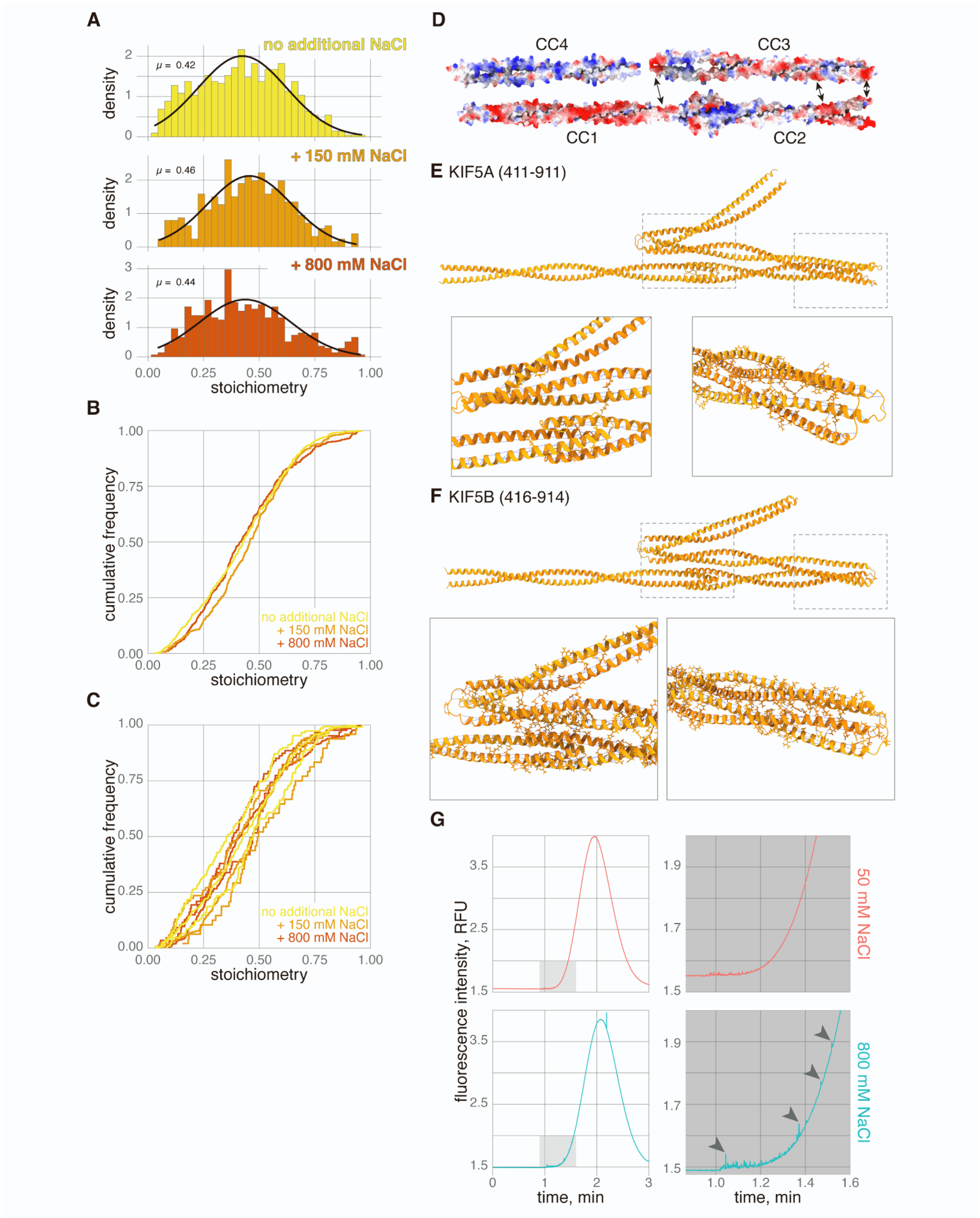

Figure S4 - Stoichiometry is insensitive to NaCl concentration, related to Figure 4.
A - C. Histograms (A), cumulative frequency plots of pooled data (B) and biological replicates (C), comparing the stoichiometry of CLIP-KIF5A-SNAP samples in 0 (yellow), 150 (orange) or 800 mM (brown) additional NaCl.
D. Electrostatic surface charges of the separate coiled-coil domains (CC1-4, as labelled) of human KIF5A. Potential positive charge clashes are highlighted by arrows. Models are the same as those in Figure S6.
E, F. AlphaFold2 predictions of the stalk domains of KIF5A (D) and KIF5B (E) with expanded regions below highlighting hydrogen bonds and electrostatic interactions that may stack the coiled coils domains against each other in Hinge 1 (right) and across CC1-4 incorporating Hinge 2 (left). Hydrogen bonding across CC1-4

is a factor positioning stacking of coiled-coils in AlphaFold predictions of the kinesin stalk. This structure is experimentally unverified, but is a consistent prediction by AlphaFold of the kinesin-1 stalks of multiple species using AlphaFold 2.3 onwards.

G. Representative Taylorgrams for CLIP-KIF5A-SNAP in 50 mM (red) or 800 mM (blue) additional NaCl. Grey box is expanded to the right to show spikes of aggregated protein, highlighted by arrowheads.

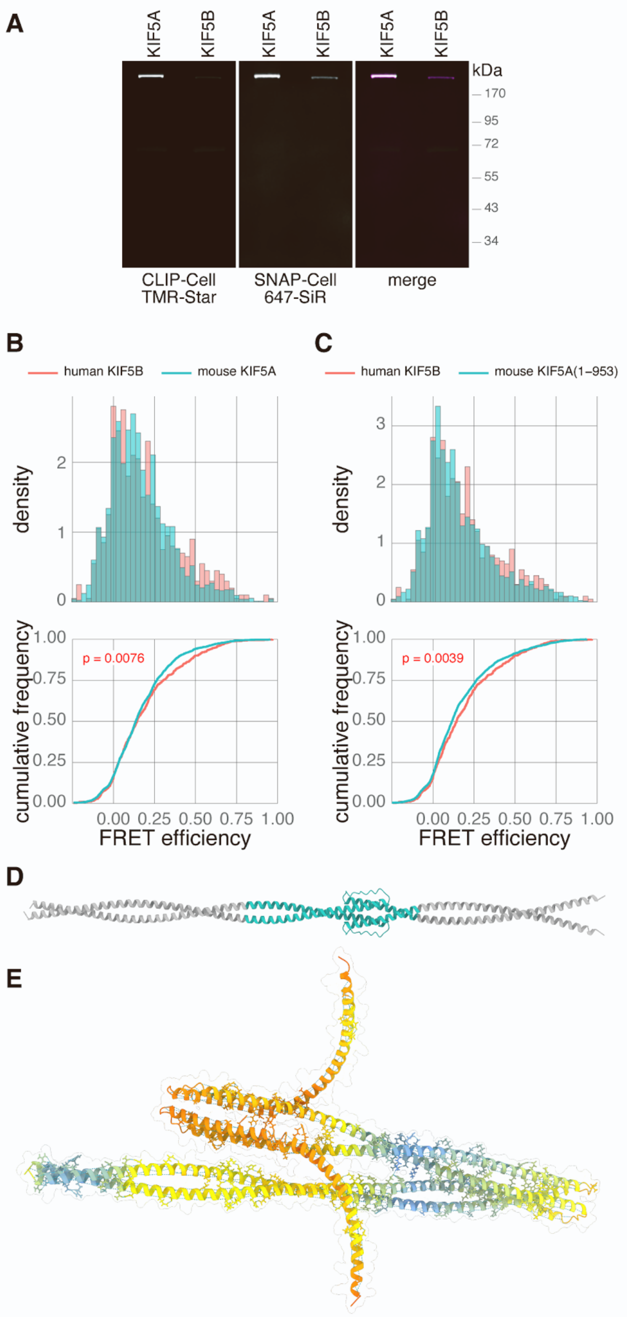

Figure S5. Consistency in FRET efficiency across Kinesin-1 isoforms, related to Figure 5.

A. In-gel fluorescence of cell lysate from HEK 293 cells transfected with CLIP-KIF5A-SNAP or CLIP-KIF5B-SNAP, and incubated with CLIP-Cell TMR-Star and SNAP-Cell 647-SiR. The merged fluorescence panel (right) shows 647-SiR in magenta and TMR-Star in green. KIF5B consistently had lower expression than KIF5A in this expression system, which may reflect tighter regulation of protein levels for an isoform expressed endogenously in HEK 293 cells.

B, C. Histograms and cumulative frequency plots comparing the FRET efficiency of human KIF5B (red) to full length mouse KIF5A (B) or KIF5A(1-953) (C), truncated to be equivalent in length to KIF5B. Full length KIF5A has a slightly higher FRET efficiency compared to KIF5B, which would be anticipated due to its longer length, whereas KIF5A(1-953) is almost identical.

D. An AlphaFold2 prediction of CC1-CC2 of KIF5A, with deleted residues 505-610 (spanning the retrograde loop), highlighted in turquoise.

E. An AlphaFold2 prediction of KIF5A tail (411-1027) with residues 505-610 deleted (residues 921-1027 are hidden for clarity). The structure is coloured by pLDDT score. The predicted structure of the deletion mutant shows that the coiled-coil structure of CC1-CC2 of the stalk is retained with deletion, but the removal of the retrograde loop removes interactions between CC2 to CC3, with knock-on effects for the positioning of CC4.

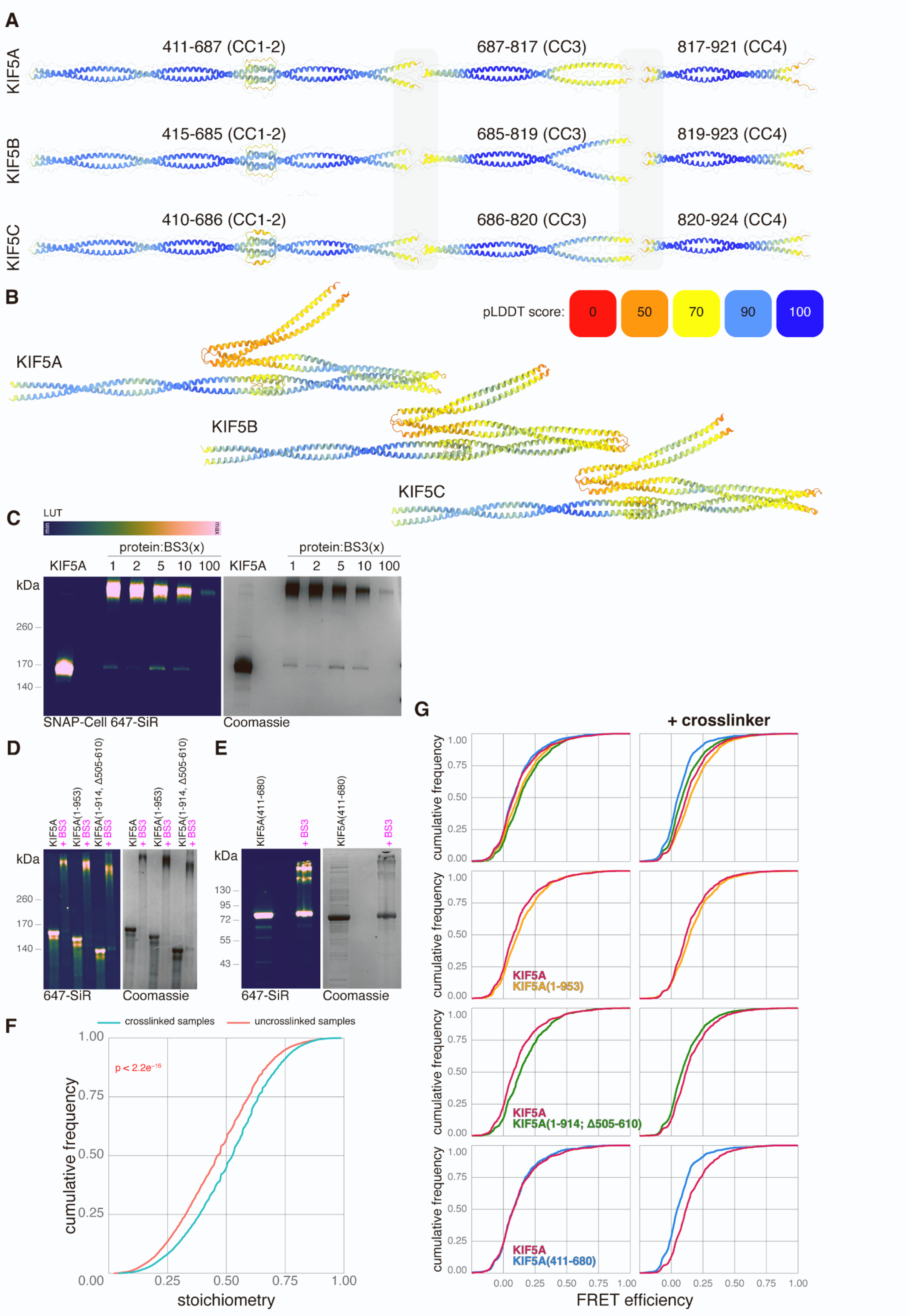

Figure S6. AlphaFold predictions of domains suggest conformational flexibility, related to Figure 6.
A, B. AlphaFold2 predictions of kinesin-1 stalk domains either individually (A) or as a complete stalk (B). Boundaries between domains for A were defined by predicted aligned errors (PAE) domains, and centred on the unstructured regions in full tail domain predictions (namely the linker, hinge 1 and hinge 2 in Figure 2B). This creates three coiled coil regions from left to right: CC1-CC2 (packed together by the retrograde loop),

CC3 and CC4. Grey boxes highlight phase breakdown of coiled-coils). Predictions illustrated in B were modelled as the complete tail domain, including the C-terminal intrinsically disordered region, but this region was omitted from the figure for clarity.

C. In-gel fluorescence of 647-SiR labelled CLIP-KIF5A-SNAP (left) and Coomassie stain of the same (right) purified from HEK 293 cells and treated with various ratios (by mass) of the crosslinking reagent BS3. The amount of BS3 is indicated above the lane and compared to the uncrosslinked sample labelled 'KIF5A'. A ratio of 1:1 was used for onward experiments, as little uncrosslinked KIF5A remained and higher amounts of BS3 trapped kinesin in the well, resulting in lower band intensities at high molecular weight. The batlow scientific colour map was applied to the fluorescent gel image to indicate intensity.

D, E. Example protein samples from Figure 6 separated by SDS-PAGE and imaged by in-gel fluorescence of 647-SiR labelled SNAP or by Coomassie stain. Full length KIF5A and mutants (as indicated, D) or a CC1-CC2 KIF5A fragment (E) purified from HEK 293 cells and optionally treated with a 1:1 ratio by mass of the crosslinking reagent BS3. The batlow scientific colour map was applied to the fluorescent gel images to indicate intensity, and is illustrated in panel C.

F. A cumulative frequency plot comparing the stoichiometry of crosslinked (blue) and uncrosslinked (red) samples. Stoichiometry always shifted to the right in crosslinked samples, strongly implying that BS3 had a tendency to quench fluorescence, particularly of the SNAP-tag.

G. Cumulative frequency plots of data in Figure 6 comparing FRET efficiencies of uncrosslinked (left) and crosslinked (right) protein samples of KIF5A (pink), KIF5A(1-953) (yellow), KIF5A(1-914; Δ505-610) (green), and KIF5A(411-680) (blue).

	Motor region				Stalk region							Tail
This work	Motor	Neck linker	CC0	ID linker	CC1	Retrograde loop	CC2	Hinge 1	CC3	Hinge 2	CC4	ID tail
Carrington et al., 2024[S3]	Motor		Neck CC	Hinge 1	CC1	Hinge 2	CC2	Elbow	CC3		CC4	unstructured
Tan et al., 2023[S4]	Motor		CC1		CC2		CC3a	Hinge	CC3b	Linker	CC4	tail
Weijman et al., 2022[S5]	Motor		CC0	Hinge 1	CC1	Hinge 2	CC2	Elbow	CC3		CC4	tail
Hackney, 2007[S6]	Motor	Neck linker	Coil-0		Coil-1		Coil-2		Coil-3		Coil-4	tail
Friedman and Vale, 1999[S7]	Motor		Neck	Hinge 1	Coil 1	Hinge 2	Coil 2					tail
Coy et al., 1999[S8]	Motor		Dimer-isation	Swivel	Coil 1	Hinge	Coil 2					tail

Figure S7. Kinesin-1 domain nomenclature, related to Figure 7.
A comparison of the naming of kinesin-1 domains over time. 'CC' stands for coiled-coil and 'ID' stands for intrinsically disordered. The variance in naming conventions reflects the shifting predictions of the kinesin-1 coiled-coil domains over time. For this reason exact domain boundaries are not listed; for example, CC2 in Hackney 2007[S6] originally extended further into what is currently known as CC3. Discrepancy also arises from whether the Neck coiled coil is numbered as '0' or '1'. Given the consensus that the unstructured region between CC1 and CC2 is unlikely to be a flexible hinge, we have renamed this the 'retrograde loop', reflecting its function of interleaving CC1 and CC2 together. Finally, to give unique identifiers to all unstructured regions in kinesin-1 we used 'linker' within the motor region and 'hinge' in the stalk region, particularly as there is a similarity in the length of hinges 1 and 2 in the stalk.

References

S1. Kaan, H.Y.K., Hackney, D.D., and Kozielski, F. (2011). The structure of the kinesin-1 motor-tail complex reveals the mechanism of autoinhibition. Science *333*, 883–885. https://doi.org/10.1126/science.1204824.

S2. Atherton, J., Chegkazi, M.S., Leusciatti, M., Di Palma, M., Peirano, E., Pozzer, L.S., Meli, M.V.A., Pasqualato, S., Foran, T., Morra, G., et al. (2025). Microtubule association induces a Mg-free apo-like ADP pre-release conformation in kinesin-1 that is unaffected by its autoinhibitory tail. Nat. Commun. *16*, 6214. https://doi.org/10.1038/s41467-025-61498-3.

S3. Carrington, G., Fatima, U., Caramujo, I., Lewis, T., Casas-Mao, D., and Peckham, M. (2024). A Multiscale approach reveals the molecular architecture of the autoinhibited kinesin KIF5A. J. Biol. Chem., 105713. https://doi.org/10.1016/j.jbc.2024.105713.

S4. Tan, Z., Yue, Y., da Veiga Leprevost, F., Haynes, S.E., Basrur, V., Nesvizhskii, A.I., Verhey, K.J., and Cianfrocco, M.A. (2023). Autoinhibited kinesin-1 adopts a hierarchical folding pattern. Elife 12, 2023.01.26.525761. https://doi.org/10.7554/eLife.86776.

S5. Weijman, J.F., Yadav, S.K.N., Surridge, K.J., Cross, J.A., Borucu, U., Mantell, J., Woolfson, D.N., Schaffitzel, C., and Dodding, M.P. (2022). Molecular architecture of the autoinhibited kinesin-1 lambda particle. Sci Adv 8, eabp9660. https://doi.org/10.1126/sciadv.abp9660.

S6. Hackney D.D. (2007). Jump-starting kinesin. J Cell Biol. 176(1), 7-9. doi: 10.1083/jcb.200611082.

S7. Friedman, D.S., and Vale, R.D. (1999). Single-molecule analysis of kinesin motility reveals regulation by the cargo-binding tail domain. Nat. Cell Biol. 1, 293–297. https://doi.org/10.1038/13008.

S8. Coy, D.L., Hancock, W.O., Wagenbach, M., and Howard, J. (1999). Kinesin's tail domain is an inhibitory regulator of the motor domain. Nat. Cell Biol. 1, 288–292. https://doi.org/10.1038/13001.
